# Influence of different pretreatments and drying methods on the chemical compositions and bioactivities of Smilacis Glabrae Rhizoma

**DOI:** 10.1186/s13020-022-00614-7

**Published:** 2022-05-06

**Authors:** Juanjuan Qiao, Gengyu Lu, Gang Wu, Hui Liu, Wanli Wang, Tianmao Zhang, Guoyong Xie, Minjian Qin

**Affiliations:** 1 Department of Resources Science of Traditional Chinese Medicines, School of Traditional Chinese Pharmacy, China Pharmaceutical University, Nanjing, 211198 China; 2 The Teaching Experiments Center of Traditional Chinese Medicines, School of Traditional Chinese Pharmacy, China Pharmaceutical University, Nanjing, 211198 China; 3 Yangzhou Center for Food and Drug Control, Yangzhou, 225000 China

**Keywords:** Smilacis Glabrae Rhizoma, Pretreatment and drying method, UHPLC-Q-TOF-MS/MS and UHPLC-DAD analysis, Astilbin stereoisomers, Bioactivities

## Abstract

**Background:**

The processing of medicinal plant materials is one of the important factors influencing the components and biological activities of TCMs. *Smilax glabra* Roxb. is an herbal vine widely distributed in China, and its dried rhizome (Smilacis Glabrae Rhizoma, SGR) is often used in traditional medicines and functional foods. The processing methods of fresh cutting for SGR slices have been included in ancient Chinese herbal works, some local standards of TCMs, and the current Chinese Pharmacopoeia. Nevertheless, to date, the scientific basis for the processing of fresh medicinal materials for SGR slices has not been revealed.

**Methods:**

To optimize the processing method for preparing SGR slices from the fresh rhizomes, the chemical compositions of the un-pretreated and pretreated (boiling, steaming) samples before and after drying (sun-drying, shade-drying, oven-drying), and the contents of astilbin isomers in dried SGR were analyzed by UHPLC-Q-TOF-MS/MS and UHPLC-DAD methods, respectively. Then, the antioxidant, anti-inflammatory, xanthine oxidase and α-glucosidase inhibitory activities of the prepared SGR slices were investigated by biological assays.

**Results:**

A total of fifty-two compounds were identified from the un-pretreated and pretreated samples and a total of forty-nine compounds were identified from the subsequently dried samples. After pretreated by boiling and steaming, the contents of neoastilbin, neoisoastilbin, and isoastilbin in the prepared samples all increased. As a quality marker of SGR, the content of astilbin was unchanged or decreased slightly compared with that in the un-pretreated samples. During the drying process, the contents of the four astilbin stereoisomers in the un-pretreated samples increased significantly, while those in the pretreated samples had a slight increase or decrease. The effects of different processing methods were sorted according to the bioactivities of the prepared SGR. As a result, SGR slices prepared with no pretreatment followed by a sun-drying process have a higher astilbin content, better bioactivities and more energy savings, representing the optimum processing method for SGR slices.

**Conclusions:**

This study reveals the scientific basis for the processing of fresh medicinal materials for SGR slices. The results provide scientific information for the quality control of SGR and its rational applications in herbal medicines and functional foods.

**Graphical abstract:**

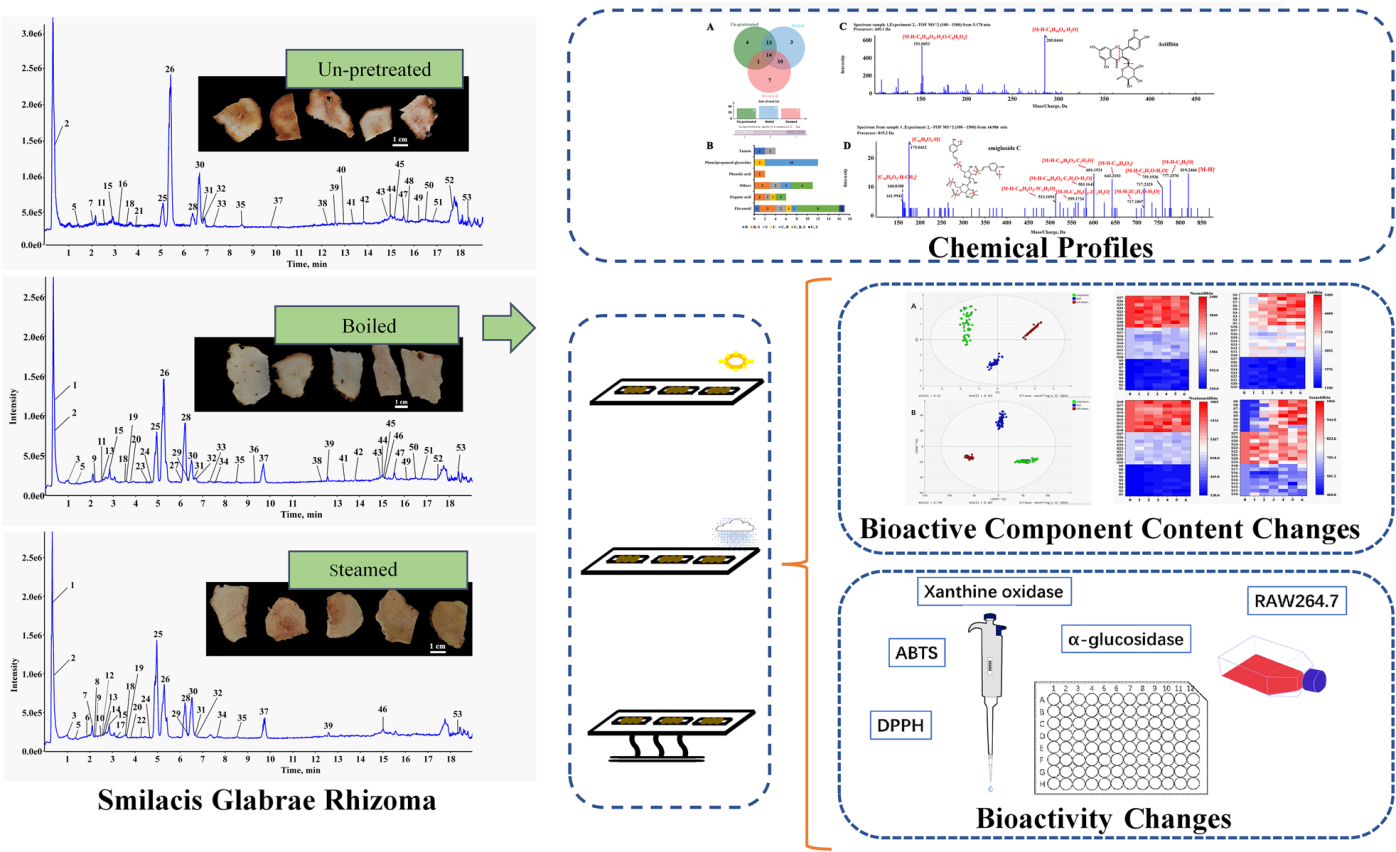

**Supplementary Information:**

The online version contains supplementary material available at 10.1186/s13020-022-00614-7.

## Background

*Smilax glabra* Roxb. (Smilacaceae) is an herbal vine that is widely distributed in China, India, Vietnam, Myanmar and Thailand [1]. The dried rhizome of *Smilax glabra* (Smilacis Glabrae Rhizoma, SGR) is often used as a traditional Chinese medicine (TCM), called “Tu Fu Ling” in Chinese. SGR is recorded in the Chinese Pharmacopoeia and is used to treat syphilis, furunculosis, eczema, dermatitis, nephritis, cystitis, and mercury poisoning [2]. SGR is also the main ingredient of “Gui Ling Gao” (turtle jelly), a well-known functional food popular in southern China [3, 4]. To date, modern scientific studies on SGR have demonstrated that it possesses multiple biological and pharmacological activities, including anti-inflammatory [5, 6], antioxidant [7], antipsoriasis [8], anticancer [9, 10], and antihyperuricemia [6] effects. In addition, according to the recent studies, SGR also exhibits strong inhibitory activity against α-glucosidase (α-Glu) [11] and has an antidiabetic effect [12].

It is generally believed that the multiple biological and pharmacological activities of TCMs are ascribed to their chemical components. The main compounds in SGR are flavonoids, phenylpropanoids and organic acids [13–16]. Flavonoids, such as astilbin and its stereoisomers are regarded as the main bioactive compounds in SGR [13, 14]. Astilbin has been used as a marker for SGR quality control according to the Chinese Pharmacopoeia [2].

Quality control of the main bioactive components is important for the safety, efficacy and consistency of TCMs and functional foods. The processing of plant materials is one of the important factors influencing their components and biological activities [17–19]. Traditionally, the processing method for SGR is very simple. The unsliced dried rhizome is washed and soaked with water and then sliced and dried. However, SGR needs a long time for soaking and rinsing before slicing, which might cause the loss of active components. Moreover, SGR is tough and fibrous, so fresh cutting of SGR is relatively easy, and the morphology of the slices changes minimally after drying. The processing methods of fresh cutting for SGR slices have been included in ancient Chinese herbal works, some local processing standards of TCMs, and the current Chinese Pharmacopoeia [2]. Nevertheless, to date, the scientific basis for the processing of fresh medicinal materials to prepare SGR slices has not been revealed [20].

Some Chinese herbal works recorded that the fresh rhizomatic materials of *S. glabra* could be pretreated by “boiling” and “steaming” and then sliced and dried [21]. However, hot water boiling of rhizomatic herbal materials could cause starch gelatinization and might inactivate glycoside enzymes in the plant materials [22, 23]. To date, no reports have indicated whether the effective ingredients and biological activities of SGR are affected by “boiling” and “steaming” pretreatments.

Drying technology is widely used in the fields of herbs and foods as an initial processing method for preservation and extending the shelf life [17, 18]. Some low-cost drying methods that are commonly used to process herbs and foods include sun-drying, shade-drying and oven-drying. As a result of different drying methods and treatment conditions, the drying process may prevent the loss of some components [24], or may cause the loss of some components. In previous studies on the processing of SGR, certain drying methods were studied, which were mainly energy-consuming drying methods [20, 25]. There was also a report about rational analysis of peeling or nonpeeling of SGR [26]. In addition, the sulfur fumigation of SGR and its effect on the quality of the herb have been studied [27]. However, the pretreatment methods recorded in some Chinese herbal works and the changes in chemical composition during the drying process have not yet been reported. The relationships between the drying methods and SGR bioactivity remain unclear.

To optimize the processing methods for SGR slices produced from the fresh rhizomes, the effects of “boiling” and “steaming” pretreatments and three drying methods, namely, sun-drying (Su.D), shade-drying (Sh.D) and oven-drying (Ov.D) treatments, on the chemical components and bioactivities of SGR were studied. The chemical profiles of pretreated and un-pretreated SGR samples and the subsequently dried samples dealt with different drying methods were analyzed and compared by ultra high-performance liquid chromatography/quadrupole time-of-flight mass spectrometry (UHPLC-Q-TOF-MS/MS). The contents of astilbin and its three stereoisomers in SGR were determined using ultra high-performance liquid chromatography coupled with diode array detection (UHPLC-DAD). The relationships between the different pretreatments and drying methods and the biological activities of the prepared dried SGR slices were also analyzed.

## Methods

### Materials and chemical reagents.

Rhizomes of *S. glabra* were collected from Lishui (Zhejiang Province, China) in November 2020 and identified by Professor Minjian Qin. The voucher specimen (No. TFL2020110501) was deposited at the Center of Herbarium, China Pharmaceutical University, Nanjing, China (Herbarium Code: CPU).

Standard reference compounds of astilbin, neoastilbin, neoisoastilbin, and isoastilbin were purchased from Chengdu Push Bio-technology Co., Ltd. (Chengdu, China), and their purity was more than 98%. 2,2′-Azino-bis (3-ethylbenzothiazoline-6-sulfonic acid) diammonium salt (ABTS, A1888), 1,1-diphenyl-2-picrylhydrazyl (DPPH), xanthine oxidase (XO, X1875), α-Glu (G5003), and lipopolysaccharide (LPS, L2880) were purchased from Sigma–Aldrich (Shanghai, China). The nitric oxide (NO) assay kit was provided by Beyotime Co., Ltd. (Shanghai, China). Chromatography grade acetonitrile and formic acid were purchased from Merck (Darmstadt, Germany) and Macklin (Shanghai, China), respectively. All the other chemicals and reagents used were of analytical grade.

### Processes for pretreatment and drying

**Fig. 1:**
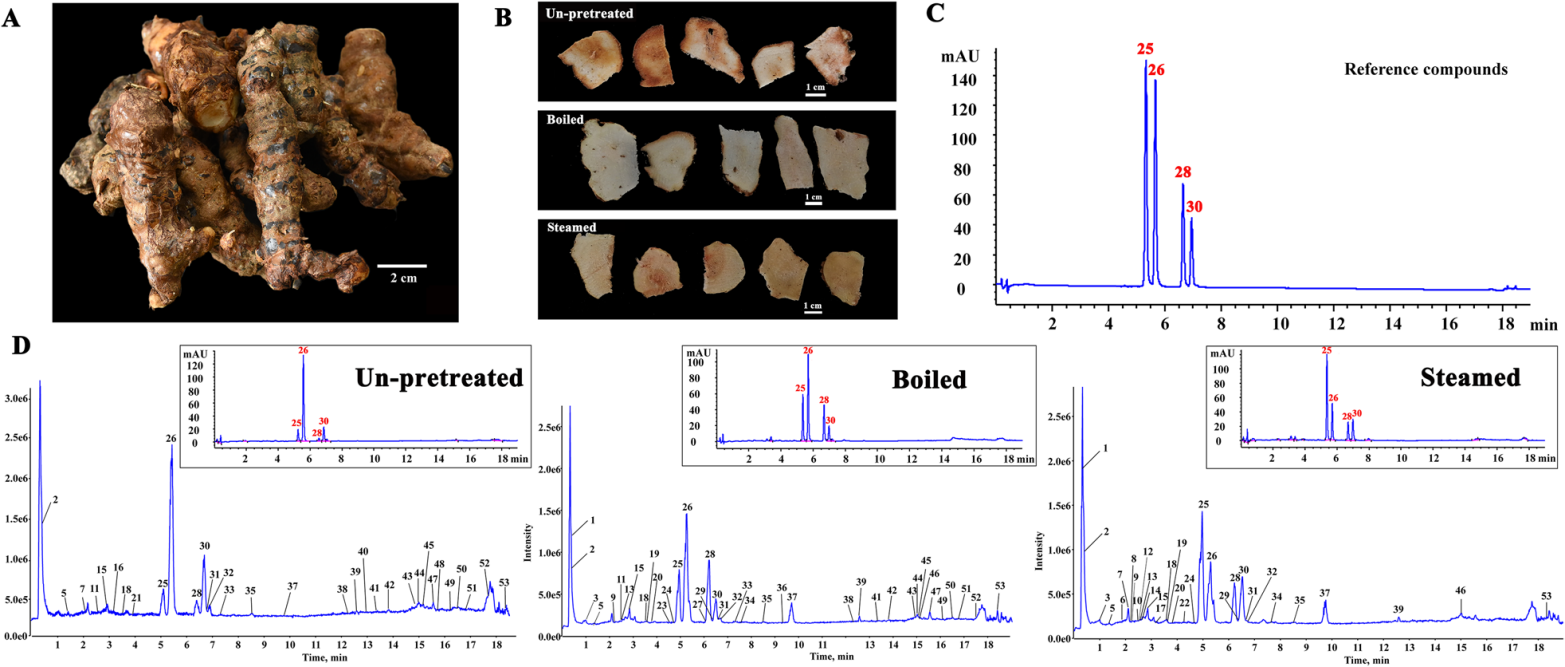
Characterization and content analysis of Smilacis Glabrae Rhizoma (SGR) **A** Fresh SGR. **B** Slices of SGR. **C** UHPLC chromatograms of reference compounds at 291 nm. (25: neoastilbin, 26: astilbin, 28: neoisoastilbin, 30: isoastilbin). **D** TIC chromatogram of three pretreated SGR samples in negative ionization mode obtained by UHPLC-Q-TOF-MS/MS and UHPLC chromatograms of SGR samples at 291 nm. (25: neoastilbin, 26: astilbin, 28: neoisoastilbin, 30: isoastilbin)

Fresh rhizomes of *S. glabra* were collected and sent to the laboratory within 24 h, and their fibrous roots were removed (Fig. 1A). Then, these fresh rhizomes were divided into three groups: in the un-pretreated group (**un-pretreated**), samples were sliced directly (with a slice thickness of 1 mm) and then dried; in the boiled group (**boiled**), samples were boiled in boiling water for 15 min prior to slicing and drying; and in the steamed group (**steamed**), samples were steamed at 121 °C for 30 min in an autoclave (GI54DS, Zealway Instrument Inc, Xiamen, Fujian, China) and then sliced and dried (Fig. 1B). The drying process included three conditions for each group: sun-drying (Su.D, dried under the sunlight, 18–20 °C, ventilation), shade-drying (Sh.D, dried in a room, 25 °C, ventilation), and oven-drying (Ov.D) at temperatures ranging from 45 °C to 105 °C at 10 °C intervals using a thermostatic oven (GZX-9246 MBE, Boxun Industrial Co., Ltd., Shanghai, China). The sampling intervals are summarized in Table 1 and Additional file 1: Table S1. At each sampling point, three aliquots (20.0 g) were randomly taken and placed in a desiccator for cooling and then pulverized in liquid nitrogen (20 mesh). The moisture content of each sample at different sampling points was measured using a digital moisture analyzer (Sartorius MA 35, Sartorius AG, Göttingen, Germany) at 105 °C according to the method established by our laboratory [28], and all assays were performed in triplicate. The remaining samples were stored at −80 °C to determine the content of bioactive components. The final sampling points of the processes were set as points at which the moisture content of the samples reached the desired standard, which is less than 15.0% according to the Chinese Pharmacopoeia [2].

**Table 1:** Sampling interval, drying efficiency of SGR (n = 3)

Drying methods	Sampling interval	Un-pretreated		Boiled		Steamed	
		No	Drying time (Final moisture %)	No	Drying time (Final moisture %)	No	Drying time (Final moisture%)
Su.D	40 min	S1	200 min (12.91)	S10	200 min (12.28)	S19	200 min (9.63)
Sh.D	4 h	S2	20 h (13.15)	S11	24 h (13.61)	S20	20 h (13.42)
Ov.D							
45 °C	40 min	S3	200 min (7.21)	S12	160 min (13.85)	S21	160 min (8.42)
55 °C	20 min	S4	100 min (11.48)	S13	100 min (10.84)	S22	100 min (8.41)
65 °C	20 min	S5	100 min (7.34)	S14	80 min (12.62)	S23	80 min (12.61)
75 °C	15 min	S6	60 min (14.41)	S15	75 min (5.61)	S24	60 min (14.29)
85 °C	12 min	S7	60 min (5.01)	S16	60 min (3.97)	S25	48 min (13.28)
95 °C	10 min	S8	50 min (4.67)	S17	50 min (5.06)	S26	40 min (12.9)
105 °C	8 min	S9	40 min (6.57)	S18	40 min (8.38)	S27	40 min (5.09)

The 189 samples prepared under different treatment conditions (three pretreatment methods combined with nine drying procedures) were used for determining the contents of four astilbin stereoisomers and the moisture content. The samples obtained at the final sampling points including 27 batches of dried SGR sliced samples were used to evaluate the antioxidant, XO inhibition, α-Glu inhibition, and anti-inflammatory activities.

### Preparation of the solution of standard reference compounds

Four standard reference compounds, namely, astilbin (1.96 mg), neoastilbin (1.94 mg), neoisoastilbin (2.06 mg), and isoastilbin (2.00 mg), were dissolved with methanol into a 1 mL volumetric flask to prepare stock solutions of the standard reference compounds. The mixed stock solution containing the four standard reference compounds was serially diluted for the construction of calibration curves and method validation.

### Extraction of bioactive compounds from the SGR samples

To optimize the conditions for the extraction of bioactive components in SGR, response surface methodology (RSM) was carried out. The methods and results are shown in Additional file 2. According to the results of the RSM study, the extraction of bioactive components from the SGR samples was performed as follows. Powder of the prepared SGR sample (0.3 g DW, 20 mesh) was placed in a 50 mL centrifuge tube and mixed with 9 mL of 71% ethanol. The mixed solution was extracted twice by an ultrasonic bath (KH-300DB, Kunshan Ultrasonic Instrument Co., Ltd., Jiangsu, China), each time for 32 min. After ultrasonic extraction, the extract was centrifuged at 10,000 rpm for 10 min. The supernatant was collected in a 25 mL brown volumetric flask and diluted to volume with 71% ethanol. The samples were filtered through a 0.22 μm microfiltration membrane before UHPLC analysis.

### UHPLC-Q-TOF-MS/MS and UHPLC-DAD conditions

UHPLC-Q-TOF-MS/MS analysis was performed on an Agilent Series 1290 UHPLC instrument (Agilent Technologies, Cambridge, MA, USA) and AB SCIEX TripleToF^®^ 4600 mass analyzer (AB SCIEX, Concord, ON, Canada) operating separately in negative and positive ion modes. Chromatography was performed at 40 °C on an Agilent ZORBAX Eclipse Plus C18 column (2.1 × 50 mm, 1.8-Micro) with a flow rate of 0.4 mL/min; the injection volume was 2 μL. The mobile phase consisted of solvents A (0.1% aqueous formic acid, v/v) and B (acetonitrile). The gradient elution program was as follows: 0–1 min, 5–7% B; 1–2 min, 7–11% B; 2–6 min, 11–16% B; 6–9.5 min, 16–18% B; 9.5–10 min, 18–18% B; 10–14 min, 18–28% B; 14–14.5 min, 28–35% B; 14.5–15.5 min, 35–35% B; 15.5–16 min, 35–40% B; 16–17 min, 40–40% B; 17–17.5 min, 40–95% B; and 17.5–19 min, 95–95% B. The following parameter settings were also used: MS survey scan of 100–1500 Da (250 ms accumulation time) and MS/MS survey scan of 100–1500 Da (100 ms accumulation time) with declustering potential of 100 V; ion source voltage, 4500 V; ion source heater, 550 °C; and collision energy, 44 V. The MS/MS data were analyzed using PeakView^®^–Analyst^®^ TF 1.6 software (AB SCIEX, Concord, ON, Canada).

The contents of astilbin and its three stereoisomers (neoastilbin, neoisoastilbin, and isoastilbin) in each SGR sample were determined using an Agilent Series 1290 UHPLC instrument (Agilent Technologies, Cambridge, MA, USA) equipped with a DAD detector (Cambridge, MA, USA). The chromatography conditions were the same as those used for UHPLC-Q-TOF-MS/MS analysis, and the DAD wavelength was set to 291 nm. Each sample was analyzed in triplicate.

### Antioxidant activity assays

For the DPPH**·** radical activity assay [29], briefly, 50 μL of a serially diluted sample solution was added to 150 μL of DPPH (75 μM). The solutions were shaken vigorously and placed in the dark at room temperature (RT) for 30 min. The absorbance (OD) was measured at 517 nm using an Epoch microplate spectrophotometer (BioTek, USA). Triplicate measurements were performed in all experiments. Ascorbic acid was used as the positive control. The DPPH**·** radical scavenging activity was calculated using the following equation:

$${\text{DPPH}}\cdot{\text{scavenging activity}}\;\% \;{ = }\;{\text{(OD}}_{control} - {\text{OD}}_{sample} )/{\text{OD}}_{control} \times 100\%$$

For the ABTS^+^ radical activity assay [30], ABTS^+^ was produced by reacting 7 mM ABTS aqueous solution with 2.45 mM potassium persulfate aqueous solution. After 12 h in the dark, the solution was diluted with PBS until the OD value was approximately 0.7 at 734 nm. The SGR sample solution (50 μL) was mixed with 150 μL of ABTS^+^ for 30 min and then measured at a wavelength of 734 nm. Triplicate measurements were performed in all experiments. Ascorbic acid was used as the positive control. The ABTS^**+**^ radical scavenging activity was calculated using the following equation:

$${\text{ABTS}}^{ + } \;{\text{scavenging activity}}\;\% \;{ = }\;\left( {{\text{OD}}_{control} - \;{\text{OD}}_{sample} } \right)/{\text{OD}}_{control} \times 100\%$$

### Enzyme inhibition assay

#### XO inhibition assay

The inhibitory activity of the SGR sample against XO was assessed spectrophotometrically, following the method of Yin Wan et al. [31], with some modifications. XO, xanthine, and 4-nitro blue tetrazolium chloride (NBT) were dissolved in phosphate buffer (50 mM, pH 7.8). A 10 μL portion of the SGR extract and 10 μL of XO (0.6 U/mL) were added to 250 μL of phosphate buffer and incubated at 37 °C for 15 min. Subsequently, the solution was mixed with 20 μL of xanthine (30 mM) and 10 μL of NBT (0.6 mM) and incubated at 37 °C for 30 min, and then the absorbance was measured with a spectrophotometer at a wavelength of 560 nm. The sample without SGR extract was used as the control and allopurinol was used as a positive control. The percentage of XO inhibition was calculated as follows:

$${\text{XO}}\;{\text{inhibition }}\;\% \;{ = }\;{\text{(OD}}_{control} - {\text{OD}}_{sample} )/{\text{OD}}_{control} \times 100\%$$

#### α-Glu inhibition assay

α-Glu inhibition was assessed according to the literature with modifications [32]. α-Glu (0.04 U/mL) and substrate *p*-nitrophenyl-α-d-glucopyranoside (PNPG, 0.4 mM) were dissolved in phosphate buffer (0.1 M, pH 6.8). Briefly, 50 μL of SGR at different concentrations was mixed with 70 μL of α-Glu solution and incubated at 37 °C for 5 min. Subsequently, 50 μL of PNPG was added to start the reaction, and then, the solution was incubated at 37 °C for 30 min. The reaction was stopped by the addition of 80 μL of Na_2_CO_3_ aqueous solution (0.2 M). After 5 min at RT, the absorbance of the sample was measured at a wavelength of 405 nm. Acarbose was used as a positive control, and α-Glu inhibitory activity was calculated using the following formula:

$$\alpha {\text{ - Glu }}\;{\text{inhibition }}\;\% \;{ = }\;{\text{(OD}}_{control} - {\text{OD}}_{sample} )/{\text{OD}}_{control} \times 100\%$$

### Anti-inflammatory activity

#### Cell culture and viability assay

RAW264.7 macrophages were obtained from the Department of Pharmacology of Traditional Chinese Medicines, China Pharmaceutical University (Nanjing, China), and were cultured in Dulbecco’s modified essential medium (DMEM, Gibco) containing 10% fetal bovine serum (FBS, Sigma–Aldrich) and penicillin and streptomycin (Biosharp) at 37 °C in a humidified 5% CO_2_ incubator (3111 type, Thermo Fisher Scientific). Cells were seeded in a 96-well culture plate at a density of 10 × 10^5^ cells/well and allowed to attach at 37 °C for 24 h [33]. Cells were treated with SGR extracts (final concentration, low (L), medium (M) and high (H) dose of 0.53, 1.06, and 2.13 mg/mL, respectively) and LPS (final concentration, 1 µg/mL) for an additional 24 h. The formazan crystals were dissolved in 150 µL of dimethyl sulfoxide (DMSO) for 15 min, and the absorbance was detected at 570 nm.

#### Determination of NO concentrations

The RAW264.7 macrophages were plated at a density of 10 × 10^5^ cells/mL and cultured for 24 h. Then, the cells were incubated with LPS (final concentration, 1 µg/mL) and SGR extracts (final concentration, low (L), medium (M) and high (H) dose of 0.53, 1.06, and 2.13 mg/mL, respectively) and cultured for 24 h at 37 °C. Then, the cell culture supernatant was collected for determination of the NO concentration using the manufacturer’s instructions. The detection wavelength was 540 nm and NaNO_2_ was used as the standard.

## Statistical analysis

Data are presented as the mean ± standard deviation, and the mean values of the analysis and cell experiment were from three and six independent replicates, respectively. Statistical analysis was performed using Microsoft Excel 2016 (Microsoft Inc., Redmond WA, USA); the IC_50_ values were calculated using GraphPad Prism 7 (San Diego, CA, USA); heatmaps were generated using Origin 2019b software (OriginLab Corporation, Northampton, MA, USA); and SIMCA 13.0 software (Umetrics, Umea, Sweden) was used for principal component analysis (PCA) and orthogonal projections to latent structures discriminant analysis (OPLS-DA).

## Results

### Drying efficiencies

The initial moisture content of the un-pretreated SGR sample was 56.49 ± 1.66%. After pretreatment, the moisture content of the boiled and steamed samples was 55.14 ± 1.61% and 53.15 ± 1.8%, respectively. This result indicated that the moisture content was slightly reduced after performing the two pretreatment methods. The dried SGR slice samples and drying efficiencies are shown in Table 1.

The Sh.D samples required 20 h to be dried, and the final moisture contents reached the desired standard of less than 15.0% according to the Chinese Pharmacopoeia. However, the Su.D and Ov.D samples took less than 3 h to be dried within a moisture content of 15.0%. The drying efficiencies of Ov.D treatment were positively correlated with the temperature.

### The contents of four astilbin stereoisomers determined by UHPLC-DAD

The contents of astilbin, neoastilbin, neoisoastilbin, and isoastilbin in the SGR samples were simultaneously determined by the UHPLC-DAD method. Validation of the UHPLC analysis method was carried out, with the established regression equations for determining the four astilbin stereoisomers shown in Table 2, and the limit of detection (LOD), limit of quantitation (LOQ), stability, precision, and reproducibility of each compound were determined (Additional file 1: Tables S2 and S3). UHPLC chromatograms of standard reference compounds are shown in Fig. 1C. All calibration curves showed good linear correlation (R^2^ > 0.9993) within the linear ranges. The results indicated that the method employed was rapid, accurate, and precise for determining the contents of the four major compounds in the SGR samples. The contents of the four astilbin stereoisomers are shown in Additional file 1: Tables S4–12.

**Table 2:** Calibration curve equations of astilbin, neoastilbin, neoisoastilbin, and isoastilbin

Peak	Rt ( min)	Compounds	Regression equation	R^2^
24	4.565	Neoastilbin	y = 12.926 × − 2.3048	0.9993
25	4.879	Astilbin	y = 12.677 × − 2.2413	0.9993
27	5.757	Neoisoastilbin	y = 11.606 × − 1.5159	0.9993
29	6.098	Isoastilbin	y = 13.034 × − 1.2695	0.9993

### Chemical profiles of the SGR samples after different pretreatments

**Fig. 2:**
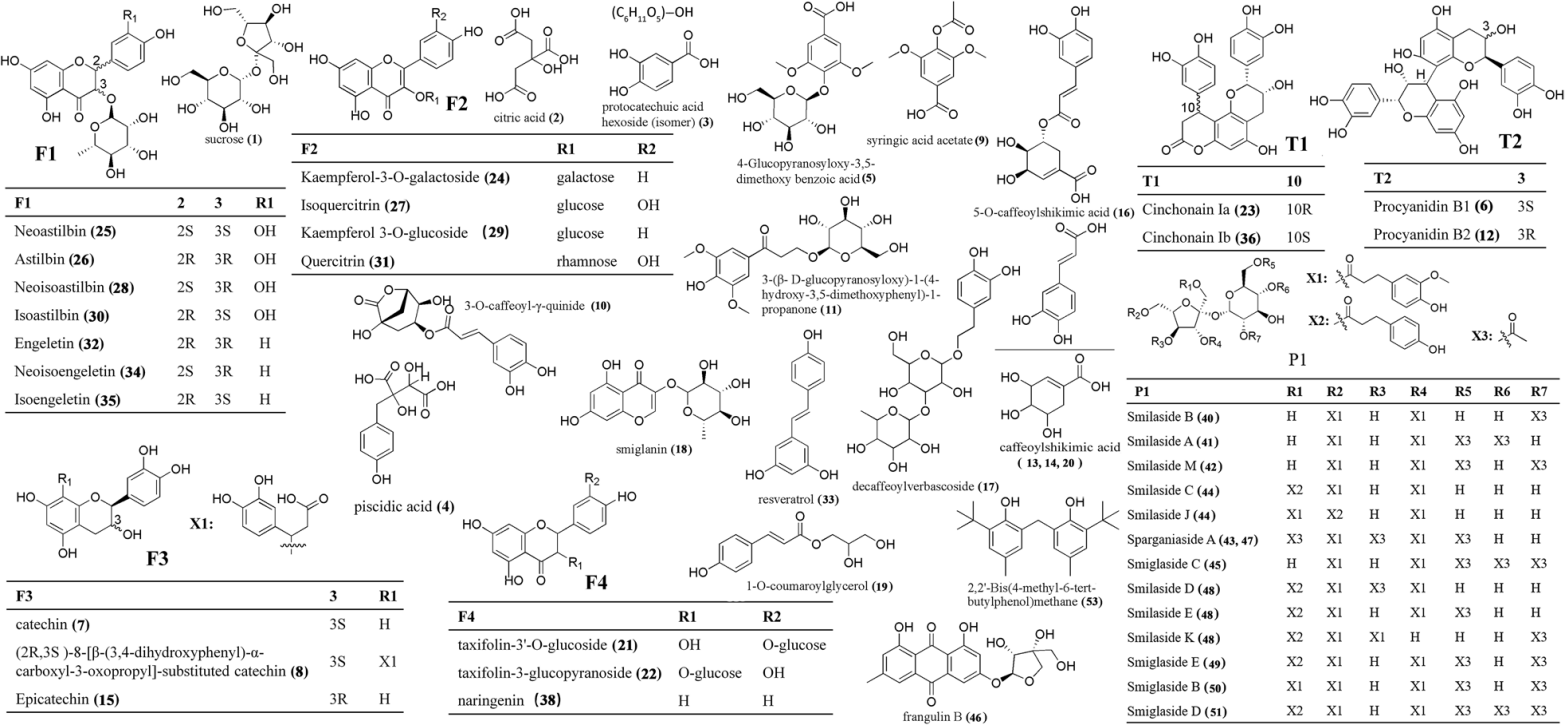
Chemical structures of the compounds identified in SGR samples

**Fig. 3:**
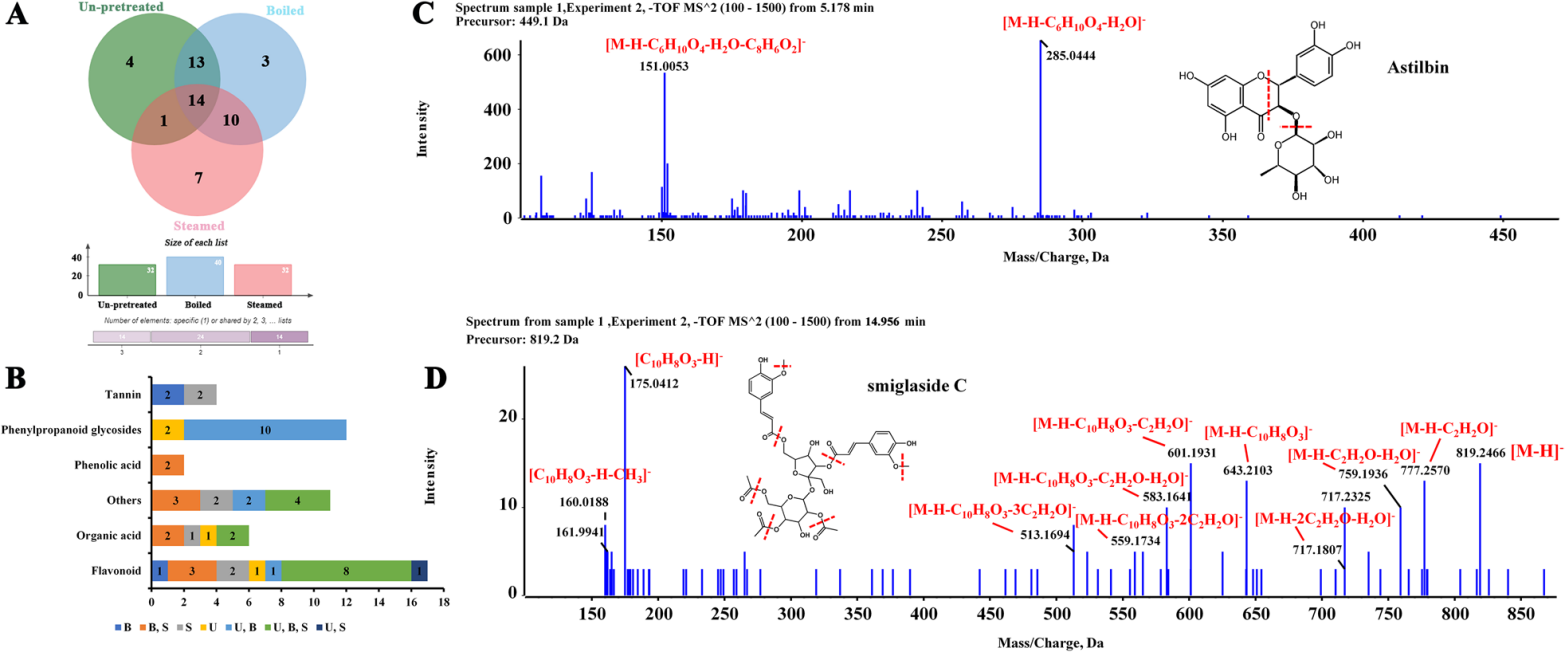
The chemical profiles of SGR after three pretreatment methods. **A** Venn diagrams of identified compounds in SGR. **B** Distribution of different compound types. (U, un-pretreated; B, boiled; S, steamed) **C** MS/MS spectra of astilbin in negative ionization mode. **D** MS/MS spectra of smiglaside C in negative ionization mode

The un-pretreated samples and the samples pretreated by boiling and steaming prior to being dried (Table 3, 0 min) were analyzed by UHPLC-Q-TOF-MS/MS. Both positive and negative ion modes were used, but the peak shape and the numbers of peaks were better in negative ion mode. The total ion chromatograms (TICs) and MS data of the extracts of un-pretreated, boiled, and steamed SGR samples in negative ESI mode are shown in Fig. 1D. A total of fifty-two compounds were detected and identified from the three groups of samples (Fig. 2, Table 3), including seventeen flavonoids, twelve phenylpropanoid glycosides, six organic acids, two phenolic acids, four tannins, and eleven other compounds. Thirty-two compounds were identified from the un-pretreated SGR samples. Forty compounds were identified from the boiled SGR samples. Thirty-two compounds were identified from the steamed SGR samples (Fig. 3A). The three groups of samples shared fourteen compounds, eight of which were flavonoids, namely, epicatechin, neoastilbin, astilbin, neoisoastilbin, isoastilbin, quercitrin, engeletin, and isoengeletin. 5-*O*-Caffeoylshikimic acid, taxifolin-3'-*O*-glucoside, smilaside B and smilaside D (or smilaside E or smilaside K) were found only in un-pretreated samples, isoquercitrin, cinchonain Ia and cinchonain Ib were found only in boiled samples, and seven compounds including procyanidin B1, decaffeoylverbascoside, (2*R*,3*S*)-8-[*β*-(3,4-dihydroxyphenyl)-*α*-carboxyl-3-oxopropyl]-substituted catechin, 3-*O*-caffeoyl-γ-quinide, procyanidin B2, caffeoylshikimic acid III, and taxifolin-3-glucopyranoside were found only in steamed group samples (Fig. 3B, Table 3). In addition, two phenolic acids (protocatechuic acid hexoside (isomer) and syringic acid acetate) were detected only in boiled and steamed samples, and ten phenylpropanoid glycosides including smilaside A, smilaside M, sparganiaside A, smiglaside C, smilaside C (or smilaside J), smiglaside E, smiglaside B, and smiglaside D were detected only in un-pretreated and boiled groups of samples.

**Table 3:** UHPLC-MS/MS chemical profiling of SGR after different pretreatments and drying

Peak no.	Rt (min)	Selected ion	Observed/ calculate mass (m/z)	Error	Formula	MS/MS fragmentation patterns	0 min	Su.D	Sh.D	Ov.D		Identification	Ref	Classification
										65℃	105℃			
1	0.333	[M-H]^−^	341.1093/341.1089	1.1	C_12_H_22_O_11_	179, 113, 101	B, S	U, B, S	U, B, S	U, B, S	U, B, S	Sucrose	[34]	Others
2	0.373	[M-H]^−^	191.0206/191.0197	4.5	C_6_H_8_O_7_	111	U, B, S	U, B, S	U, B, S	U, B, S	U, B, S	Citric acid	[35]	Organic acid
3	0.967	[M-H]^−^	315.0726/315.0722	1.5	C_13_H_16_O_9_	152, 109, 108	B, S	U, B, S	U, B, S	U, B, S	U, B, S	Protocatechuic acid hexoside (isomer)	[36–38]	Phenolic acid
4	1.037	[M-H]^−^	255.0519/255.051	3.4	C_11_H_12_O_7_	179,165,125,107		U, B, S	U, B, S	U, B, S	U, B, S	piscidic acid	[39]	Organic acid
5	1.36	[M-H]^−^	359.098/359.0984	2	C_15_H_20_O_10_	197, 182, 153, 138, 123	U, B, S	U, B, S	U, B, S	U, B, S	U, B, S	4-glucopyranosyloxy-3,5-dimethoxy benzoic acid	[40]	Organic acid
6	1.845	[M-H]^−^	577.1378/577.1352	4.6	C_30_H_26_O_12_	425, 407, 381, 289, 245, 125	S	U, B, S	U, B	B, S	U, B, S	Procyanidin B1	[37]	Tannin
7	2.111	[M-H]^−^	289.0722/289.0718	1.5	C_15_H_14_O_6_	271, 245, 205, 179, 151	U, S					Catechin	[14, 35]	Flavonoid
8	2.256	[M-H]^−^	469.1147/469.114	1.4	C_24_H_22_O_10_	315, 289, 247, 205, 109	S	B, S	B, S	U, B, S	U, B, S	(2*R*,3*S*)-8-[*β*-(3,4-dihydroxyphenyl)-*α*-carboxyl-3-oxopropyl]-substituted catechin	[14, 41]	Flavonoid
9	2.262	[M-H]^−^	239.0556/239.0561	− 2.1	C_11_H_12_O_6_	149, 133, 107	B, S	U, B, S	U, B, S	U, B, S	U, B, S	Syringic acid acetate	[42]	Phenolic acid
10	2.445	[M-H]^−^	335.0783/335.0772	3.1	C_16_H_16_O_8_	291, 179, 161, 135	S	S	S			3-*O*-caffeoyl-γ-quinide	[43]	Others
11	2.556	[M-H]^−^	387.1290/387.1297	-1.7	C_17_H_24_O_10_	207, 192, 177	U, B	U, B, S	U, B, S	U, B, S	U, B, S	3-(*β*-d-glucopyranosyloxy)-1-(4-hydroxy-3,5-dimethoxyphenyl)-1-propanone	[14]	Others
12	2.579	[M-H]^−^	577.1375/577.1352	4	C_30_H_26_O_12_	425, 407, 381, 289, 245, 125	S	U, B, S	U, B, S	S	U, B, S	Procyanidin B2	[37]	Tannin
13	2.626	[M-H]^−^	335.0760/335.0772	− 3.6	C_16_H_16_O_8_	291, 179, 161, 135	B, S	B, S				(Trans) caffeoylshikimic acid II	[44]	Organic acid
14	2.748	[M-H]^−^	335.0765/335.0772	− 2.1	C_16_H_16_O_8_	291, 179, 161, 135	S	B, S	U, B, S	U, B, S	U, B, S	Caffeoylshikimic acid III	[44]	Organic acid
15	2.874	[M-H]^−^	289.0716/289.0718	− 0.5	C_15_H_14_O_6_	205, 201, 187, 151, 123, 109	U, B, S	U, B, S	U, B, S	U, B, S	U, B, S	Epicatechin	[35]	Flavonoid
16	3.078	[M-H]^−^	335.0775/335.0772	0.8	C_16_H_14_O_8_	291, 179, 161, 135	U	U, B	U, B, S	U, B, S	U, B, S	5-*O*-caffeoylshikimic acid	[14, 45]	Organic acid
17	3.134	[M-H]^−^	461.1687/461.1665	4.8	C_20_H_30_O_12_	415, 191, 149, 113	S	U, B, S	U, B, S	U, B, S	U, B, S	Decaffeoylverbascoside	[46]	Others
18	3.582	[M-H]^−^	339.0730/339.0722	2.5	C_15_H_16_O_9_	193, 192	U, B, S	U, B, S	U, B, S	U, B, S	U, B, S	Smiglanin	[14, 47]	Others
19	3.608	[M-H]^−^	237.0770/237.0769	0.6	C_12_H_14_O_5_	145,119,117	B, S	U, B, S	U, B, S	U, B, S	U, B, S	1-*O*-coumaroylglycerol	[48]	Others
20	3.817	[M-H]^−^	335.0780/335.0772	2.3	C_16_H_16_O_8_	291, 179, 161, 135	B, S	U, B, S	U, B, S	U, B, S	U, B, S	Caffeoylshikimic acid IV	[44]	Organic acid
21	3.899	[M-H]^−^	465.1047/465.1039	1.9	C_21_H_22_O_12_	437, 303, 285, 179, 151	U	U, B	U, B, S	U, B, S	U, B, S	Taxifolin-3'-*O*-glucoside	[36]	Flavonoid
22	4.218	[M-H]^−^	465.1045/465.1039	1.4	C_21_H_22_O_12_	303, 285, 275, 151	S					Taxifolin-3-glucopyranoside	[49]	Flavonoid
23	4.774	[M-H]^−^	451.1050/451.1034	3.4	C_24_H_20_O_9_	341	B	U, B, S	U, B, S	U, B, S	U, B, S	Cinchonain Ia	[41]	Tannin
24	4.925	[M-H]^−^	447.0931/447.0933	− 0.5	C_21_H_20_O_11_	283, 255, 239, 211	B, S	U	U, B	S		Kaempferol-3-*O*-galactoside	[50]	Flavonoid
25	4.952	[M-H]^−^	449.1111/449.1089	4.9	C_21_H_22_O_11_	303, 285, 151	U, B, S	U, B, S	U, B, S	U, B, S	U, B, S	Neoastilbin	[14]	Flavonoid
26	5.183	[M-H]^−^	449.1098/449.1089	1.9	C_21_H_22_O_11_	303, 285, 151	U, B, S	U, B, S	U, B, S	U, B, S	U, B, S	Astilbin	[45]	Flavonoid
27	6.194	[M-H]^−^	463.0865/463.0882	− 3.7	C_21_H_20_O_12_	299, 271, 255, 243, 231	B		B			Isoquercitrin	[51]	Flavonoid
28	6.201	[M-H]^−^	449.1099/449.1089	2.1	C_21_H_22_O_11_	303, 285, 151	U, B, S	U, B, S	U, B, S	U, B, S	U, B, S	Neoisoastilbin	[14]	Flavonoid
29	6.225	[M-H]^−^	447.0947/447.0933	0.9	C_21_H_20_O_11_	285, 255, 239, 179, 159	B, S		U, B, S	U	B	Kaempferol 3-*O*-glucoside	[50, 52]	Flavonoid
30	6.457	[M-H]^−^	449.1099/449.1089	2.1	C_21_H_22_O_11_	303, 285, 151	U, B, S	U, B, S	U, B, S	U, B, S	U, B, S	Isoastilbin	[14, 36]	Flavonoid
31	6.634	[M-H]^−^	447.0947/447.0933	3.2	C_21_H_20_O_11_	301, 283, 255, 239, 179, 151	U, B, S	U	U, B	U, B	U, B	Quercitrin	[53]	Flavonoid
32	6.658	[M-H]^−^	433.1151/433.1140	2.5	C_21_H_22_O_10_	287, 269, 259, 178, 152	U, B, S	U, B, S	U, B, S	U, B, S	U, B	Engeletin	[14, 45, 52]	Flavonoid
33	7.297	[M-H]^−^	227.0722/227.0714	− 4.7	C_14_H_12_O_3_	185, 183, 159, 157, 143	U, B	U, B, S	U, B, S	U, B, S	U, B, S	Resveratrol	[14, 52, 54]	Others
34	7.615	[M-H]^−^	433.1143/433.1140	0.6	C_21_H_22_O_10_	287, 269, 259, 180, 152	B, S	B, S	B, S	B, S	B, S	Neoisoengeletin	[14, 52]	Flavonoid
35	8.466	[M-H]^−^	433.1147/433.1140	1.6	C_21_H_22_O_10_	287, 269, 259, 180, 152	U, B, S	U, B, S	U, B, S	U, B, S	U, B, S	Isoengeletin	[14, 52]	Flavonoid
36	9.389	[M-H]^−^	451.1023/451.1034	− 2.6	C_24_H_20_O_9_	341	B	U, B, S	U, B, S	U, B, S	U, B, S	Cinchonain Ib	[41, 52]	Tannin
37	9.59	[M + CH_3_COOH-H]^−^	723.5067/723.5053	2	C_41_H_72_O_10_	677, 451, 225	U, B, S					Unknown		Others
38	12.393	[M-H]^−^	271.0620/271.0612	3	C_15_H_12_O_5_	177, 151, 119, 107	U, B	U, B, S	U, B, S	U, B, S	U, B, S	Naringenin	[14]	Flavonoid
39	12.521	[M-H] ^+^	453.3441/453.3422	4.2	C_23_H_50_O_8_	569, 343, 326, 227, 209, 111, 100	U, B, S					Unknown		Others
40	13.11	[M-H]^−^	735.2151735.2142	1.2	C_34_H_40_O_18_	559, 499, 193, 175, 160	U	U, B, S	U, B, S	U, B, S	U, B, S	Smilaside B	[36]	Phenylpropanoid glycosides
41	13.413	[M-H]^−^	777.2269/777.2247	2.8	C_36_H_42_O_19_	735, 717, 601, 559, 499, 193, 175, 160	U, B	U, B, S	U, B, S	U, B, S	U, B, S	Smilaside A	[36, 55]	Phenylpropanoid glycosides
42	13.834	[M-H]^−^	777.2282/777.2247	4.4	C_36_H_42_O_19_	735, 717, 601, 559, 193, 175, 160	U, B	U, B, S	U, B, S	U, B, S	U, B, S	Smilaside M	[36, 56]	Phenylpropanoid glycosides
43	14.922	[M-H]^−^	819.2362/819.2353	1.0	C_38_H_44_O_20_	777, 759, 583, 559, 193, 175, 160	U, B	U, B, S	U, B, S	U, B, S	U, B, S	Sparganiaside A or Phenylpropanoid glycosides	[14, 15, 36]	Phenylpropanoid glycosides
44	14.939	[M-H]^−^	839.2393/839.2404	− 1.3	C_41_H_44_O_19_	693, 663, 645, 483, 319, 217, 175, 145	U, B	U, B, S	U, S	U, B, S	U, B, S	Smilaside C or smilaside J	[36]	Phenylpropanoid glycosides
45	14.956	[M-H]^−^	819.2383/819.2353	3.6	C_38_H_44_O_20_	777, 717, 601, 583, 513, 175	U, B	U, B, S	U, B, S	U, B, S	U, B, S	Smiglaside C	[36]	Phenylpropanoid glycosides
46	15.046	[M-H]^−^	401.0897/401.0878	4.7	C_20_H_18_O_9_	313, 121	B, S	U, B, S	U, B, S	U, B, S	U, B, S	Frangulin B	[57]	Others
47	15.483	[M-H]^−^	819.2364/819.2353	1.3	C_38_H_44_O_20_	777, 643, 601, 583, 513, 175	U, B	B, S	U, B, S	U, B, S	U, B, S	Sparganiaside A or phenylpropanoid glycosides	[14, 15, 36]	Phenylpropanoid glycosides
48	15.58	[M-H]^−^	881.2553/881.2510	4.9	C_43_H_46_O_20_	735, 705, 687, 559, 483, 175	U	U, B, S	U, B, S	U, B, S	U, B, S	Smilaside D or smilaside E orsmilaside K	[36]	Phenylpropanoid glycosides
49	16.115	[M-H]^−^	923.2647/923.2615	3.4	C_45_H_48_O_21_	881, 863, 777, 747, 483	U, B	U, B, S	U, B, S	U, B, S	U, B, S	Smiglaside E	[15, 36]	Phenylpropanoid glycosides
50	16.463	[M-H]^−^	953.2762/953.2721	4.3	C_46_H_50_O_22_	911, 777, 759, 601	U, B	U, B, S	U, B, S	U, B, S	U, B, S	Smiglaside B	[14, 15, 36]	Phenylpropanoid glycosides
51	16.85	[M-H]^−^	965.2769/965.2721	5	C_47_H_50_O_22_	923, 905, 789, 747, 483	U, B	U, B, S	U, B, S	U, B, S	U, B, S	Smiglaside D	[14, 15, 36]	Phenylpropanoid glycosides
52	17.592	[M-H]^−^	965.2726/965.2721	0.5	C_47_H_50_O_22_	923, 905, 789, 777, 747, 559, 483, 175	U, B	U, S	U, B, S	U, B, S	U, B, S	Phenylpropanoid glycosides	[36]	Phenylpropanoid glycosides
53	18.32	[M-H]^−^	339.2344/339.2330	4.2	C_23_H_32_O_2_	163	U, B, S	U, B, S	U, B, S	U, B, S	U, B, S	2,2'-Bis(4-methyl-6-tert-butylphenol)methane	[58]	Others

U, un-pretreated; B, boiled; S, steamed; 0 min, the samples prior to being dried; Su.D, sun-drying; Sh.D, shade-drying; Ov.D 65 ℃, oven-drying at 65 ℃; Ov.D 105 ℃, oven-drying at 105 ℃

Flavonoids are the main components of SGR; kaempferol-3-*O*-galactoside (**24**), isoquercitrin **(27),** kaempferol 3-*O*-glucoside (**29**) and quercitrin (**31**) are flavonols, naringenin (**38**) is a flavanone, and the other twelve flavonoids are flavanonols. Most of the identified flavonoids belong to the *O*-glycosyl types of flavonoid glycosides, except four flavonoid aglycones (compounds **7**, **8**, **15**, and **38**). For flavonoid *O*-glycosides, including compounds **21, 22, 24, 25, 26, 27, 28, 29, 30, 31, 32, 34** and **35**, the most characteristic fragmentation behavior was C-O bond cleavage, which produced the fragments [M-H-162]^−^ or [M-H-146] ^−^ due to the losses of glucose (162 Da) and galactose (162 Da) or rhamnose (146 Da). The four main flavanonols (compounds **25**, **26**, **28** and **30**) were unambiguously identified by the same deprotonated ions at *m/z* 449 (C_21_H_21_O_11_) and the same product ions at *m/z* 285.0444 and *m/z* 151.0053, indicating that they are stereoisomers. The fragment ion at *m/z* 285.0444 corresponded to a loss of rhamnose and H_2_O from the [M-H]^−^ moiety. The reverse Diels–Alder (RDA) reaction of the C-ring also generated characteristic fragmentation of flavonoids, and the fragment ion at *m/z* 151.0053 corresponded to a loss of C_8_H_6_O_2_ from the [M-H-C_6_H_10_O_4_-H_2_O]^−^ moiety. The fragmentation is shown in the Fig. 3C. By comparison with the literature and the standard reference compounds, astilbin and its stereoisomers were identified.

Phenylpropanoid glycosides existed only in the un-pretreated and boiled groups of samples and shared similar fragmentations. For example, compound **45** gave an [M-H]^−^ ion at *m/z* 819.2364 (C_38_H_43_O_20_) in negative ion mode. The fragment ion at *m/z* 777.2570 corresponded to the loss of an acetyl group (C_2_H_2_O) from the [M-H]^−^ moiety, the fragment ion at *m/z* 759.1936 corresponded to the loss of H_2_O from 777.2570, the fragment ion at *m/z* 601.1931 corresponded to the loss of a phenyl propyl fragment (C_10_H_8_O_3_) and acetyl group (C_2_H_2_O) from the [M-H]^−^ moiety, the fragment ion at *m/z* 513.1452 corresponded to the loss of a phenyl propyl fragment (C_10_H_8_O_3_) and three acetyl groups (3C_2_H_2_O) from [M-H]^−^ moiety, and the fragment ion at *m/z* 175.0412 corresponded to a loss of H from the phenyl propyl fragment (C_10_H_8_O_3_) (Fig. 3D). Therefore, the fragment ions of compound **45** corresponded to the successive or simultaneous losses of acetyl, methyl, H_2_O, phenyl and propyl moieties. By comparison with the literature[36], compound **45** was tentatively identified as smiglaside C. Compounds **43** and **47** showed the same molecular ion at *m/z* 819.2364 (C_38_H_43_O_20_) and same MS/MS fragmentation and were tentatively identified as sparganiaside A or the other phenylpropanoid glycoside compounds. Similarly, compounds **41, 42, 44, 49, 50, 51** and smiglaside C were different in the number and substitution position of acetyl, methyl, phenyl and propyl groups, which were tentatively identified as smilaside A, smilaside M, smilaside C (or smilaside J), smiglaside E, smiglaside B and smiglaside D, respectively.

In this study, four tannins were identified. Compounds **6** and **12** were found only in the steamed group of samples and showed the same molecular ion at *m/z* 577.1352 (C_30_H_25_O_12_) in negative ionization mode, characterized by MS/MS ions at *m/z* 425.1055, 407.0860, and 289.0800. The fragment ion at *m/z* 425.1055 corresponded to a loss of C_8_H_8_O_3_ (152 Da) from the [M-H]^−^ moiety, the fragment ion at *m/z* 407.0860 corresponded to a loss of C_8_H_10_O_4_ (170 Da) from the [M-H]^−^ moiety, and the fragment ion at *m/z* 289.0800 (C_15_H_13_O_6_) corresponded to a loss of C_15_H_12_O_6_ (288 Da) from the [M-H]^−^ moiety. The fragment ions at *m/z* 245.0858, 205.0501 and 179.031 indicated the existence of a catechin (C_15_H_14_O_6_) moiety, which implied that compounds **6** and **12** were dimers of catechin. Based on their retention time and literature data, compounds **6** and **12** were identified as procyanidin B1 and procyanidin B2, respectively. Compounds **23** and **36** were found only in the boiled group samples and showed the same molecular ion at *m/z* 451.1034 (C_24_H_19_O_9_) in negative ionization mode, characterized by MS/M*S* ions at *m/z* 341.0637, which corresponded to a loss of C_6_H_6_O_2_ (110 Da) from the [M-H]^−^ moiety. Based on their retention time and literature data, compounds **23** and **36** were identified as cinchonain Ia and cinchonain Ib, respectively.

### Influence of different pretreatments on the contents of astilbin stereoisomers in SGR

**Fig. 4:**
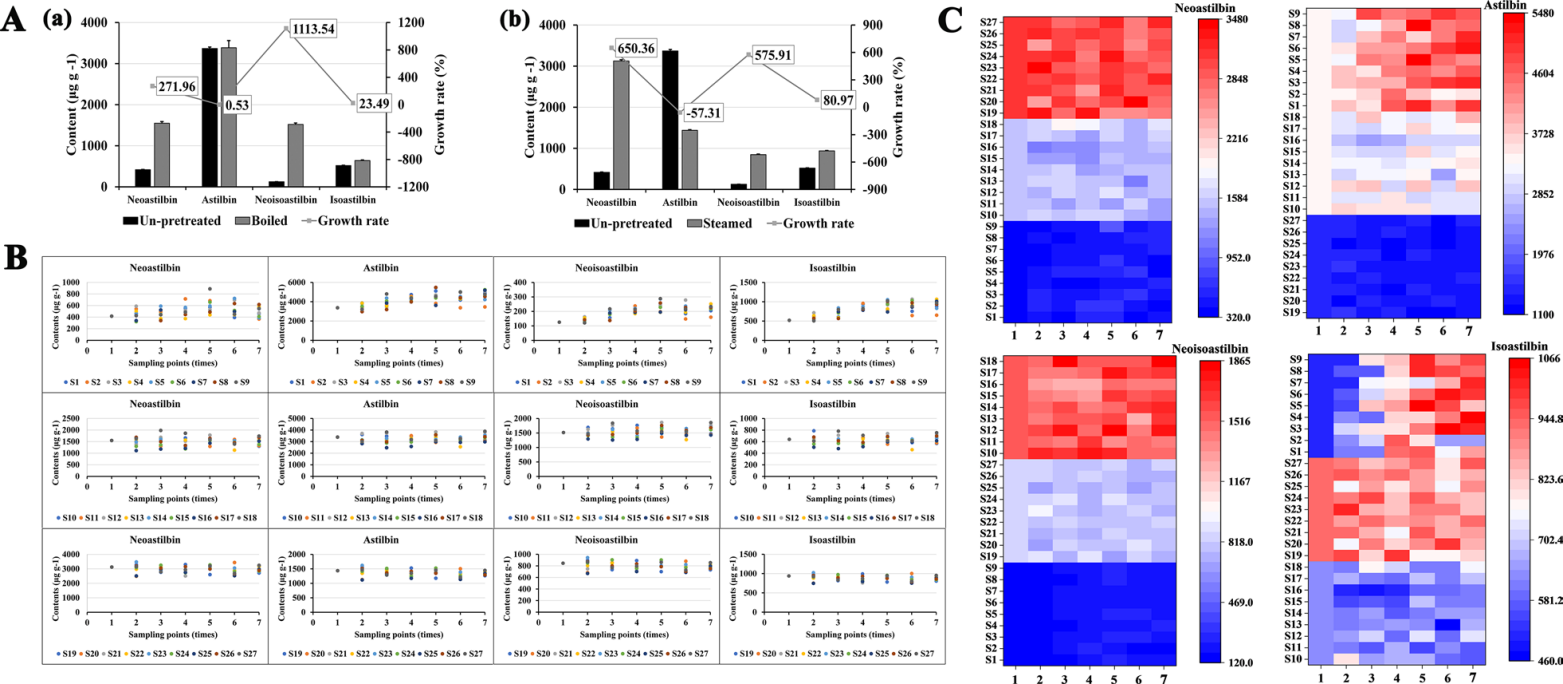
Content changes of the four astilbin isomers in SGR samples prepared by different methods. **A** Changes in the contents of the four astilbin isomers in SGR by boiling (**a**) and steaming (**b**) pretreatments compared with un-pretreated SGR samples. **B** Scatter plot of the contents (μg/g) of the four astilbin isomers throughout the drying process at different sampling times. **C** Heatmaps of the contents (μg/g) of the four astilbin isomers throughout drying process at different sampling times

Before the drying process, the contents of astilbin and its three stereoisomers in the pretreated SGR samples changed significantly compared with those in the un-pretreated fresh sample. The contents of neoastilbin, neoisoastilbin, and isoastilbin all increased after pretreatment with “boiling” and “steaming”, while the astilbin content either remained the same as that of the un-pretreated sample or decreased (Table 4, Fig. 4A).

**Table 4:** The content changes of the four astilbin isomers in SGR samples pretreated by different methods

Pretreatment methods	Neoastilbin		Astilbin		Neoisoastilbin		Isoastilbin	
	(μg/g)	(%)	(μg/g)	(%)	(μg/g)	(%)	(μg/g)	(%)
Un-pretreated	416.5 ± 6.96	–	3368.36 ± 35.51	–	125.05 ± 1.55	–	518.45 ± 7.18	–
Boiled	1549.23 ± 37.85	+ 271.96	3386.11 ± 174.17	+ 0.53	1517.53 ± 36.45	+ 1113.54	640.24 ± 13.12	+ 23.49
Steamed	3125.3 ± 29.42	+ 650.36	1437.91 ± 14.52	− 57.31	845.22 ± 16.88	+ 575.91	938.22 ± 10.29	+ 80.97

In the boiled and steamed samples, the neoastilbin content increased by 271.96% (Fig. 4Aa) and 650.36% (Fig. 4Ab), respectively; the neoisoastilbin content increased by 1113.54% and 575.91%; and the isoastilbin content increased by 23.49% and 80.97%. In contrast, the content of astilbin remained almost unchanged in the boiled samples and decreased by 57.31% in the steamed samples.

### Influence of drying methods on the contents of astilbin stereoisomers in SGR

The contents of astilbin stereoisomers in SGR prepared by different drying methods are shown in Additional file 1: Table S13. During the drying process, the content of neoastilbin gradually decreased in the steamed group of samples. The neoastilbin content of the boiled group of samples fluctuated during the drying process and increased slightly after drying, while the change in neoastilbin content in the un-pretreated group of samples first increased and then decreased (Fig. 4B) but did not appear obvious compared with the pretreated SGR due to the small amounts (Fig. 4C).

Astilbin is a dominant bioactive compound in SGR, and it has also been found in many other herbs, fruits, and plant-based foods [59]. The astilbin content was the highest in the un-pretreated SGR group of samples, followed by the boiled group samples, and the steamed group samples had the lowest content (Fig. 4B, Fig. 4C). During the drying process, the astilbin content in the un-pretreated group of samples increased from 3368.36 ± 35.51 μg/g to the maximum content, 5264.62 ± 97.45 μg/g DW (Ov.D treatment at 75 °C), increasing by approximately 50%. The un-pretreated group of samples under Su.D or Ov.D treatment at 45 °C and 85 °C also showed better astilbin-increasing effects. The astilbin content in the boiled and the steamed groups of samples showed fluctuating changes and increased or decreased slightly with the drying process.

The content of neoisoastilbin in the boiled group samples was higher than that in the steamed group samples, while the content in the un-pretreated group samples was the lowest. During the drying process, in the un-pretreated group samples, the neoisoastilbin content increased from 125.05 ± 1.55 μg/g DW to the maximum amount, 255.25 ± 5.54 μg/g DW (Ov.D at 55 °C), increasing by approximately 104% (Fig. 4B). Because of the relatively small amounts, the changes in neoisoastilbin content in the un-pretreated group samples were not obvious (Fig. 4C), while the boiled and steamed groups of samples showed a fluctuating increase.

The isoastilbin content in the un-pretreated and two pretreated groups of samples before drying was different (Figs. 4B, C). During the drying process, in the un-pretreated group samples, the isoastilbin content increased significantly, and the content of some samples even increased by 80%; for example, the content in samples subjected to Ov.D treatment at 55 °C increased from 518.45 ± 7.18 μg/g DW to 1070.25 ± 27.55 μg/g DW. Both the boiled and steamed groups of samples showed fluctuating changes in the isoastilbin content during the drying processes, but the final content in the dried samples barely changed compared with that before drying. Prior to drying of the pretreated samples, the initial order of the isoastilbin content was steamed > boiled > un-pretreated, and after the samples were dried, the order of the isoastilbin content was un-pretreated > steamed > boiled.

### Influence of drying methods on the chemical profiles of the SGR slices

**Fig. 5:**
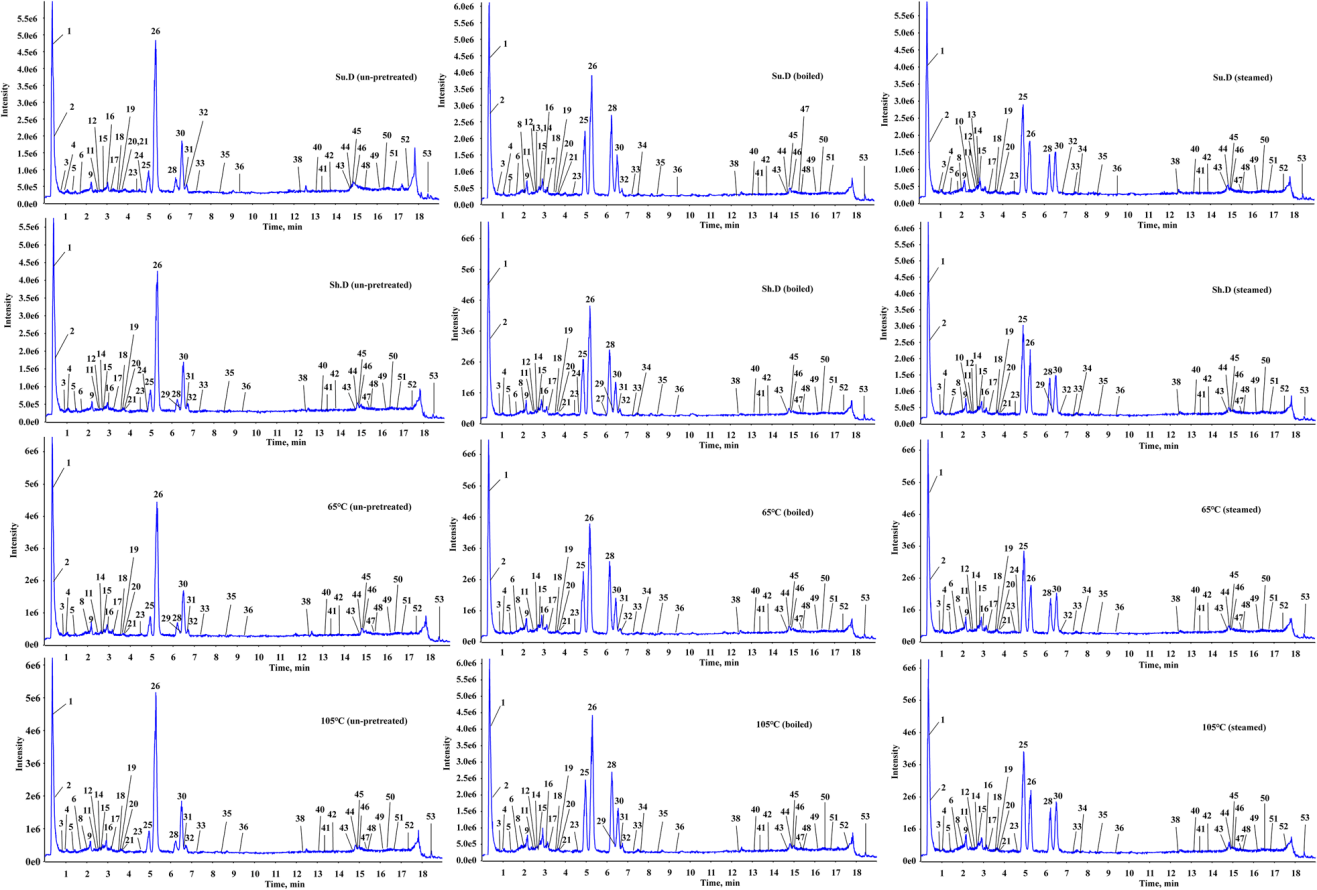
TIC chromatogram of dried SGR samples (sun-drying, shade-drying, and oven-drying at 65 °C and 105 °C) in negative ionization mode obtained by UHPLC-Q-TOF-MS/MS

UHPLC-MS/MS method was also used to analyze the chemical profiles of SGR slices obtained by three drying methods (sun-drying, shade-drying and oven-drying at 65 °C and 105 °C). As shown in Table 3 and Fig. 5, a total of 49 compounds were identified from the dried samples. All the compounds were presented in the samples before drying except piscidic acid which was newly detected after drying. As mentioned above, the chemical compositions of the undried samples with different pretreatments were obviously different and only 14 compounds were common among the samples. However, in the sun-drying, shade-drying, oven-drying at 65 °C and 105 °C samples, there were 36, 40, 40 and 41 compounds common among the samples with different pretreatments, respectively. Some compounds could be detected in all three pretreatment samples after drying which were only detected in one or two pretreatment samples before drying. In particular, phenylpropanoid glycosides were common in the un-pretreated and boiled samples before drying, but all of them can also be detected in the steamed samples after drying. Therefore, the chemical compositions of the samples after drying tended to be consistent. Previous studies have shown that different pretreatment methods have a certain impact on the content of total phenols in thyme [60]. It could be interred that the pretreatment methods in the present study changed the content of chemical components in SGR, especially the isomers of astilbin [61–63]. Moreover, degradation of the cell wall and membrane function during drying process might lead to an increase in the extractability of the active compounds in the samples [64]. Thus, the compounds with low content in the samples before drying could be detected after drying.

### Comprehensive analysis of the influences of pretreatment and drying methods on the bioactive components contents in SGR

**Fig. 6:**
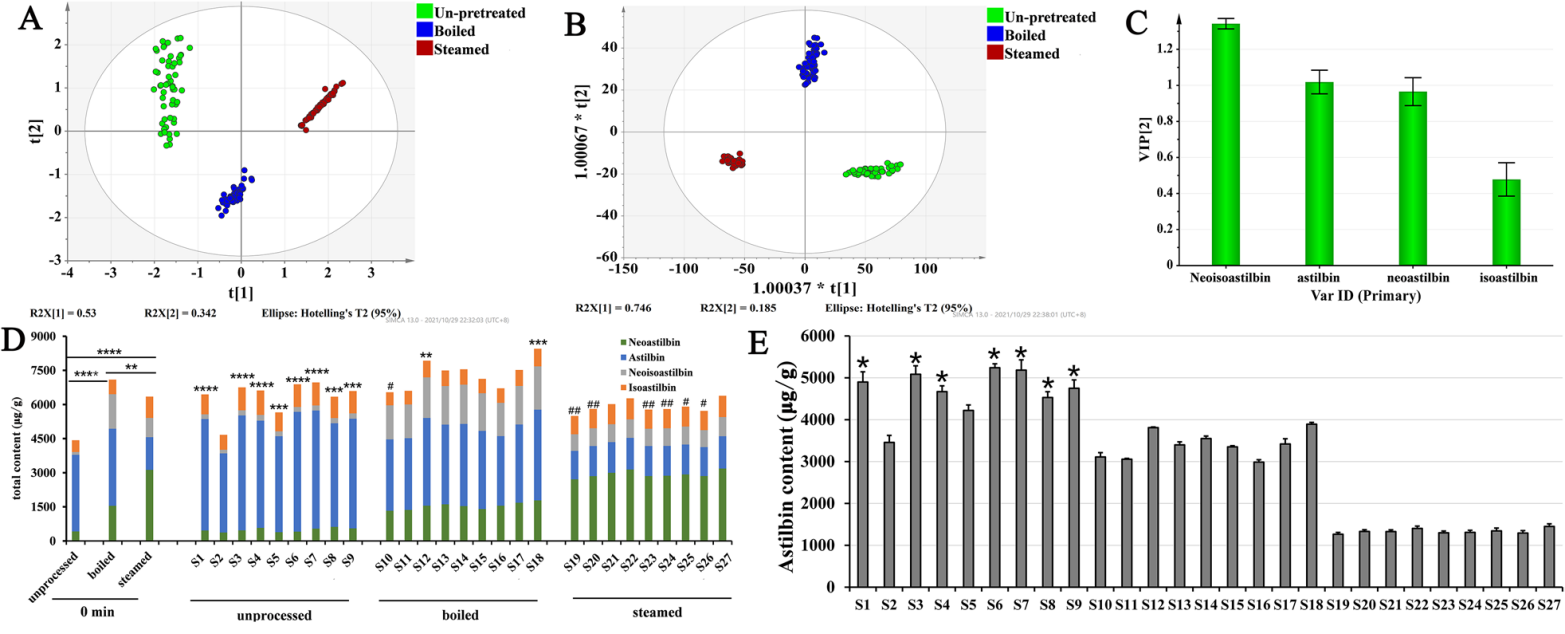
Content changes of bioactive compounds in the prepared SGR samples. **A** PCA score plot for SGR at different sampling times. **B** OPLS-DA score plot for SGR at different sampling times. **C** VIP plot for SGR at different sampling times. **D** The total contents of the four isomers under different pretreatment and drying conditions (Compared with those at 0 min, significantly increased contents were described as *, and significantly decreased contents were described as #). **E** The astilbin content of the prepared SGR samples. (*means that the astilbin content of the prepared SGR sample meets the requirement of the Chinese Pharmacopoeia)

We conducted PCA of the content data of the four astilbin stereoisomers for the 189 samples from the experimental design (Fig. 6A). In the PCA score plot of SGR slice samples, the samples could be separated into three clusters corresponding to the pretreatment methods. The values of the established PCA model fit parameters *R*^*2*^*X* (cum) and *Q*^*2*^ (cum) were 0.998 and 0.987, respectively, which indicated that the model is robust. To further investigate the potential differential compounds, sample content data were then subjected to supervised discriminant analysis, OPLS-DA. The results showed that the OPLS-DA model exhibited good fitness (*Q*^*2*^ (cum) 0.978) and predictability (*R*^*2*^*X* (cum) 0.931, *R*^*2*^*Y* (cum) 0.98). As presented in Fig. 6B, the slice samples are clearly distinguished. The variable importance in projection (VIP) scores (Fig. 6C) generated from OPLS-DA indicated the contents of neoisoastilbin and astilbin (VIP ≥ 1) conducive to distinguishing groups of SGR slice samples.

This result indicated that the pretreatment methods had significant influences on the contents of the four astilbin stereoisomers in the prepared SGR slice samples, which determined the content ratio of the four individual astilbin stereoisomers in these SGR slices. The results showed that the ratios of the contents of neoastilbin, astilbin, neoisoastilbin and isoastilbin to the total content of the four astilbin stereoisomers were 7.17%, 76.02%, 3.18%, and 13.63%, respectively, in the un-pretreated SGR sample; the ratios in the boiled sample were 21.84%, 47.74%, 21.39% and 9.03%, respectively, and those of the steamed sample were 49.24%, 22.66%, 13.32% and 14.78%. This result indicated that pretreatment changed the dominant astilbin stereoisomers in SGR. In the un-pretreated and boiled SGR samples, astilbin was dominant, while after steaming, neoastilbin became the dominant constituent in SGR.

There was a significant difference in the total content of the four astilbin stereoisomers in the un-pretreated and two pretreated groups of samples before drying (Fig. 6D). The order of the three groups of samples in terms of the total contents of astilbin stereoisomers was boiled > steamed > un-pretreated. The trend of the total contents of astilbin stereoisomers after drying was in the order boiled > un-pretreated > steamed. During the drying process, the total contents of the four astilbin stereoisomers in the un-pretreated sample group all significantly increased with decreasing moisture content, and those in the boiled and steamed sample groups slightly increased or decreased.

However, in the present study, we found that only seven treatment conditions in the un-pretreated group could meet the requirement of the Chinese Pharmacopoeia that the content of astilbin be no less than 0.45% DW (Fig. 6E).

### Antioxidant activities

**Fig. 7:**
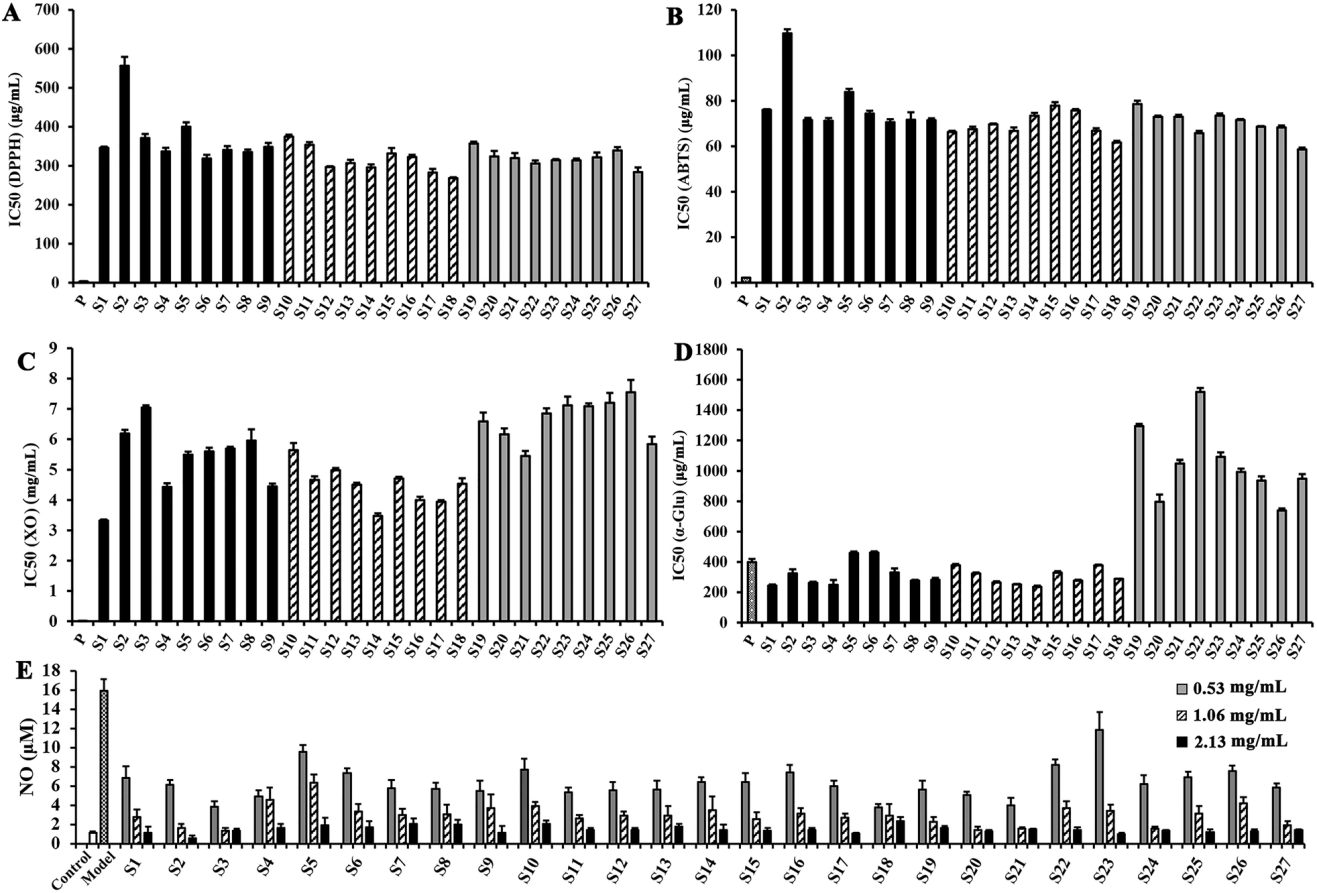
Bioactivities of the dried SGR samples under different pretreatments and drying conditions. **A** IC_50_ of the DPPH scavenging ability of SGR under different pretreatment and drying conditions. **B** IC_50_ of the ABTS^+^ scavenging ability of SGR under different pretreatment and drying conditions. **C** IC_50_ of XO inhibitory ability of SGR under different pretreatment and drying conditions. **D** IC_50_ of α-Glu inhibitory ability of SGR under different pretreatment and drying conditions. **E** Effects of different amounts of dried SGR on suppressing the production of NO (n = 6) when RAW264.7 cells were stimulated by LPS

The DPPH**·** radical scavenging ability of SGR samples prepared by the three pretreatment methods showed little change (Fig. 7A); however, the activity of SGR samples was inferior to that of the positive control drug V_C_ (IC_50_ = 3.32 ± 0.03 μg/mL). Except for the Sh.D samples in the un-pretreated group, the other SGR samples possessed strong DPPH radical scavenging abilities. Changing the drying method had a slight impact on the antioxidant activity of SGR.

The trends in ABTS^**+**^ radical scavenging ability of the SGR samples (Fig. 7B) were similar to those of the DPPH radical scavenging ability; however, the ABTS^**+**^ radical scavenging ability values were closer to that of the positive drug V_C_ (IC_50_ = 2.15 ± 0.03 μg/mL).

### Enzyme inhibition

SGR is used to treat gout in traditional Chinese medicines, and the XO inhibition is an important biochemical index to evaluate its therapeutic effect. The XO inhibitory effects of the SGR samples under different treatment conditions varied greatly (Fig. 7C). The XO inhibitory effects of the steamed group samples were generally poor. The un-pretreated SGR group samples had better effects than those of the steamed group. Overall, the XO inhibitory effects of the boiled SGR group samples were the best among the three groups of samples, while the Su.D samples of the un-pretreated group had the best effect. However, the XO inhibitory activities of the SGR samples were weaker than that of the positive control allopurinol (IC_50_ = 15.54 ± 0.35 μg/mL).

The results of in vitro bioactivity assays demonstrated that all SGR samples obtained from different pretreatment and drying methods showed good α-Glu inhibitory activities. Almost all of the IC_50_ values of the un-pretreated and boiled samples were less than that of the positive control drug acarbose (IC_50_ = 398.20 ± 21.63 μg/mL). However, the α-Glu inhibitory activities of the steamed group of samples were significantly worse than those of the un-pretreated and boiled samples. There were no significant differences in α-Glu inhibitory activities between the un-pretreated and boiled group samples, both of which had strong α-Glu inhibitory activities (Fig. 7D).

### Anti-inflammatory activity

The concentration of NO was measured to explore and compare the anti-inflammatory activities of different dried SGR samples. After treatment with SGR at concentrations of 0.53, 1.06, and 2.13 mg/mL, the viability of RAW264.7 cells exceeded 90%. As shown in Fig. 7E, the anti-inflammatory activities of steamed group samples were slightly worse than those of the other two groups of samples. Overall, the prepared SGR samples showed an obvious treatment effect on suppressing the production of NO when RAW264.7 cells were stimulated by LPS. Therefore, the results of low-concentration SGR treatment were chosen for subsequent analysis.

### Comprehensive evaluation of pretreatments and drying methods for preparing SGR slices

In this study, the biological activities of SGR under different pretreatment and drying conditions were investigated. According to the literature, astilbin and its three stereoisomers have strong antioxidant, XO and α-Glu inhibitory activities with little difference [65]. Therefore, the total content of astilbin stereoisomers showed a close correlation with the bioactivity of SGR. Additionally, it has been reported that other polar constituents in SGR have stronger XO [66] and α-Glu [67–71] inhibitory activities than astilbin stereoisomers and phenylpropanoid glycosides. This might be one of the reasons for the lower bioactivities of the steamed group samples than those of the other two groups of samples.

Entropy weight and technique for order preference by similarity to ideal solution (TOPSIS) analysis are widely used in various fields for decision-making or evaluation [72], such as in engineering, environmental applications, and other fields where precision is required [73–75]. In this study, according to the results of the entropy weight and TOPSIS analysis based on the antioxidant, XO and α-Glu inhibitory, and anti-inflammatory activities (Additional file 1: S14, Table S15), the samples of boiled SGR dried at 65 °C and un-pretreated SGR dried in the sun ranked the first and second, respectively. The un-pretreated SGR samples dried in the shade ranked 20th, indicating that the effect of shade drying is far inferior to that of sun drying. Furthermore, the Chinese Pharmacopoeia requests that the content of astilbin be at least 0.45% in SGR [2], and the un-pretreated sun-dried SGR has a higher astilbin content, better activity and more energy savings. The results further confirmed the rationality of this traditional processing method for SGR. Therefore, no pretreatment of SGR followed by drying in the sun represents the optimum treatment and drying method for preparing SGR slices from the fresh rhizomatic material of *S. glabra*.

## Discussion

SGR is often used in traditional medicines and functional foods, and SGR slices have been included in ancient Chinese herbal works, some local processing standards of TCMs, and the current Chinese Pharmacopoeia. In this research, the scientific basis for the processing of fresh medicinal materials for SGR slices was revealed. After pretreatment with “boiling” and “steaming”, the contents of the compounds changed greatly, while the chemical profiles were slightly changed compared with those of un-pretreated SGR. During the subsequent drying process, the contents of the four astilbin isomers in the un-pretreated SGR increased significantly under the action of enzymes, while the boiled and steamed SGR showed a slight increase or decrease, respectively. The boiled SGR with different drying conditions had a richer chemical composition, higher total content of four astilbin stereoisomers and strong biological activities. But according to the requirement of the Chinese Pharmacopoeia that the astilbin content be no less than 0.45% DW, only seven un-pretreated dried SGR samples were qualified medicinal materials, and the un-pretreated SGR sample also had strong biological activities. Based on the TOPSIS analysis results, no pretreatment followed by a sun-drying process was the optimum treatment and drying method for preparing SGR slices.

After “boiling” and “steaming” pretreatments, the content of neoastilbin increased significantly, because this compound is the most stable of the four stereoisomers [62]. The “boiling” and “steaming” pretreated SGR samples showed a great change in the content ratio of the four individual astilbin stereoisomers during pretreatment, which might be due to the special metabolism variation of the plant response to the stress resistance and the influences of physical and chemical factors [76–78]. The “boiling” and “steaming” pretreated SGRs retained some vitality at the beginning of the treatment, and the high temperature increased the amount of flavonoids and phenolic compounds [78, 79]. However, as the treatment time was prolonged, particular enzymes in the samples were inactivated, and the biochemical reaction of the plant itself was blocked. It has been reported that the four astilbin stereoisomers in the ethanol extract of SGR can interconvert through a chalcone intermediate, and the ratio of trans (neoastilbin and astilbin) to cis (neoisoastilbin and isoastilbin) stereoisomers (trans/cis ratio of astilbin isomers) can reach an equilibrium under certain physical and chemical conditions [62, 63]. It is deduced that the four astilbin stereoisomers in the “nonliving” prepared SGR slice could also interconvert through chalcone intermediates and finally reach an equilibrium between the amount of trans- and cis- astilbin stereoisomers. In this study, in the steamed sample, the content ratio between neoastilbin and isoastilbin was approximately 3.3:1, and that between astilbin and neoisoastilbin was approximately 1.7:1. These content ratios were close to previously reported results in which that the stable content ratio between neoastilbin and isoastilbin was 3.4:1, and that between astilbin and neoisoastilbin was 1.6:1 under incubation of an ethanol extract of SGR in a water bath at 80 °C [63]. During the drying process, the trans/cis ratio of the astilbin isomer in un-pretreated group samples varied and decreased significantly, while those in the “nonliving” steamed group samples remained almost unchanged, and the ratio of the boiled group samples decreased slightly (Additional file 1: Fig. S1). It is implied that the content of cis isomers of astilbin might increase at a faster rate (%) during the pretreatment and drying processes of SGR. This possibility provides a certain basis for the optimization of pretreatment and drying methods that are conducive to the accumulation of active components of SGR.

It is speculated that the un-pretreated rhizome of *S. glabra* remained physiologically alive after harvest and that the enzymes involved in the biosynthetic pathways of the compounds were also active [79]. During the drying process, un-pretreated rhizomes may experience a series of stress resistance reactions. These environmental stresses including solar radiation, high temperature and dewatering might induce its physiological and biochemical responses, and many reactive oxygen species (ROS) might be produced. Excess ROS might cause oxidative stress, which can result in injury to plants at both the molecular and cellular levels [80]. To avoid oxidative injury to the plant itself, ROS might induce many biochemical reactions through the action of enzymes occurring in the plant, resulting in an increased amount of flavonoids and phenolic compounds that might be used as antioxidants [76, 77]. Thus, excess ROS amounts might be particularly scavenged by antioxidant metabolites of plants [78].

Hyperuricemia and diabetes are both prevalent lifestyle-related diseases, and hyperuricemia is also a predictive factor for the development of type 2 diabetes mellitus (T2DM) [81, 82]. Hyperuricemia promotes the occurrence and development of diseases by regulating molecular signals, such as the inflammatory response, oxidative stress, and insulin resistance/diabetes [83]. Furthermore, SGR has been demonstrated to possess multiple biological and pharmacological activities, including anti-inflammatory [5, 6], antioxidant [7], antihyperuricemia [6] and antidiabetic [12] effects. To better evaluate the different pretreatment and drying methods, the antioxidant, anti-inflammatory, and XO and α-Glu inhibitory activities of SGR with different pretreatments and drying conditions were studied. This research provided data on the activities of SGR under different pretreatment and drying conditions. However, more in vivo activity differences under different processing conditions need to be further investigated.

In this study, pretreatment methods had significant effects on the content of bioactive components of SGR, which further affected the chemical characteristics of SGR. The content of astilbin in the un-pretreated SGR was the highest, the total content of the four isomers in boiled SGR was the highest, and the content of neoastilbin in steamed SGR was the highest. The antioxidant and anti-inflammatory activities of SGR in the un-pretreated and pretreated SGR were not significantly different, while the XO and α-Glu inhibitory activities of steamed SGR were weaker than those of boiled and un-pretreated SGR. It is noteworthy that there were no qualified medicinal materials before the drying of SGR, which showed that the processing of fresh SGR was an important means to achieve qualified SGR slices. However, drying in the shade, as a common drying method in the production area, is the most unfavorable drying method for the accumulation of astilbin. The Chinese Pharmacopoeia notes that SGR should be “thinly sliced while fresh, dried” and does not specify specific drying conditions. In the actual drying process, drying in the shade is a very common drying method. Therefore, we suggest that the Chinese Pharmacopoeia should clarify that SGR should not be dried in the shade. No pretreatment followed by a sun-drying process was the optimum treatment and drying method for preparing SGR slices. This conclusion has guiding significance for processing and drying in the production area.

## Conclusion

In this paper, the influences of different pretreatments and drying methods on the chemical compositions and bioactivities of Smilacis Glabrae Rhizoma were systematically studied for the first time. The chemical profiles and contents of the four individual astilbin stereoisomers were markedly changed by different pretreatments. After the “boiling” and “steaming” pretreatments, the contents of neoastilbin, neoisoastilbin, and isoastilbin all increased, while the astilbin content either remained the same as that in the un-pretreated sample or decreased. During the drying process, the contents of the four astilbin stereoisomers in the un-pretreated group of samples were all significantly increased; in particular, the content of astilbin, the quality marker of SGR, increased by approximately 50%. However, the content in the boiled and steamed groups of samples showed a slight increase or decrease.

The prepared SGR samples under different drying conditions exhibited strong antioxidant, XO and α-Glu inhibitory activities. It is noteworthy that the α-Glu inhibition activities of the un-pretreated and boiled groups of samples were significantly better than that of the positive control acarbose.

These results suggested that no pretreatment followed by a sun-drying process was the optimum treatment and drying method for preparing SGR slices. This study provides scientific information for the quality control of SGR and its rational applications in herbal medicines and functional foods.

## Supplementary Information

**Additional file 1: Table S1.** Sampling schedules during drying process of pretreated SGR. **Table S2.** The results of precision and reputability tests. **Table S3.** The results of linear ranges, LODs, LOQs and recovery tests. **Table S4.** Content of moisture and analytes in SGR during sun-drying. **Table S5.** Content of moisture and analytes in SGR during shade-drying. **Table S6.** Content of moisture and analytes in SGR during oven-drying at 45 °C. **Table S7.** Content of moisture and analytes in SGR during oven-drying at 55 °C. **Table S8.** Content of moisture and analytes in SGR during oven-drying at 65 °C. **Table S9.** Content of moisture and analytes in SGR during oven-drying at 75 °C. **Table S10.** Content of moisture and analytes in SGR during oven-drying at 85 °C. **Table S11.** Content of moisture and analytes in SGR during oven-drying at 95 °C. **Table S12.** Content of moisture and analytes in SGR during oven-drying at 105 °C. **Table S13.** Bioactive ingredient content and DPPH**·**, ABTS**·**^**+**^, XO and α-Glu(n = 3) inhibitory effects of the 27 dried SGR. **Table S14.** The operation steps and equations of entropy weight and TOPSIS analysis. **Table S15** Comprehensive evaluation result of entropy weight and TOPSIS model. **Figure S1.** The content rations of trans to cis isomers of SGR throughout drying processing at different sampling times.

**Additional file 2: S1.** Optimization of Extraction Processing for Bioactive Ingredients. **Table S1**. The Box-Behnken design experiment scheme. **Table S2**. ANOVA of quadratic model terms of three factor variables on responses. **Table S3**. Predicted and experimental values of the responses obtained under the optimal extraction conditions. **Figure S1**. Result of the single factor experiments. **Figure S2**. Response surface plots corresponding to the desirability function.

## Data Availability

Not applicable.

## Abbreviations

SGR: Smilacis Glabrae Rhizoma
Su.D: Sun-drying
Sh.D: Shade-drying
Ov.D: Oven-drying
UHPLC-Q-TOF-MS/MS: Ultra high-performance liquid chromatography coupled with electrospray ionization quadrupole time-of-flight tandem mass spectrometry
UHPLC: Ultra high-performance liquid chromatography
DAD: Diode array detector
RSM: Response surface methodology
PCA: Principal component analysis
OPLS-DA: Orthogonal projections to latent structures discriminant analysis
DMSO: Dimethyl sulfoxide
DPPH: 1,1-D-diphenyl-2-picrylhydrazyl
LPS: Lipopolysaccharide
ABTS: 2,2′-Azino-bis(3-ethylbenzothiazoline-6-sulfonic acid) diammonium salt
XO: Xanthine oxidase
α-Glu: α-Glucosidase
NO: Nitric oxide
TOPSIS: Technique for order preference by similarity to ideal solution
