# Supplementary material for: Influence of different pretreatments and drying methods on the chemical compositions and bioactivities of Smilacis Glabrae Rhizoma
Source: Chin Med. 2022 May 6;17:54. doi: 10.1186/s13020-022-00614-7 (PMC9074193; doi:10.1186/s13020-022-00614-7)
Supplement: Supplementary file 1 — Additional file 1: Table S1. Sampling schedules during drying process of pretreated SGR. Table S2. The results of precision and reputability tests. Table S3. The results of linear ranges, LODs, LOQs and recovery tests. Table S4. Content of moisture and analytes in SGR during sun-drying. Table S5. Content of moisture and analytes in SGR during shade-drying. Table S6. Content of moisture and analytes in SGR during oven-drying at 45 °C. Table S7. Content of moisture and analytes in SGR during oven-drying at 55 °C. Table S8. Content of moisture and analytes in SGR during oven-drying at 65 °C. Table S9. Content of moisture and analytes in SGR during oven-drying at 75 °C. Table S10. Content of moisture and analytes in SGR during oven-drying at 85 °C. Table S11. Content of moisture and analytes in SGR during oven-drying at 95 °C. Table S12. Content of moisture and analytes in SGR during oven-drying at 105 °C. Table S13. Bioactive ingredient content and DPPH·, ABTS·+, XO and α-Glu(n = 3) inhibitory effects of the 27 dried SGR. Table S14. The operation steps and equations of entropy weight and TOPSIS analysis. Table S15 Comprehensive evaluation result of entropy weight and TOPSIS model. Figure S1. The content rations of trans to cis isomers of SGR throughout drying processing at different sampling times. [file 13020_2022_614_MOESM1_ESM.docx]

**Influence of different pretreatments and drying methods on the chemical compositions and bioactivities of Smilacis Glabrae Rhizoma**

Juanjuan Qiao^1^, Gengyu Lu^1^, Gang Wu^2^, Hui Liu^3^, Wanli Wang^1^, Tianmao Zhang^1^, Guoyong Xie^1,^* and Minjian Qin^1,^*

^*^Correspondence: guoyongxie321@163.com; minjianqin@163.com

^1^ Department of Resources Science of Traditional Chinese Medicines, School of Traditional Chinese Pharmacy, China Pharmaceutical University, Nanjing 211198, China; kfq6310@163.com (JJQ); njlugengyu@126.com (GYL); wanliw23@163.com (WLW)

^2^ The Teaching Experiments Center of Traditional Chinese Medicines, School of Traditional Chinese Pharmacy, China Pharmaceutical University, Nanjing 211198, China; woosmail@163.com (GW)

^3^ Yangzhou Center for Food and Drug Control, Yangzhou 225000, China; liu_huiabc@163.com (HL)

**Table S1.** Sampling schedules during drying process of pretreated SGR.

| Drying methods | | Sampling times | | | | | | |
| --- | --- | --- | --- | --- | --- | --- | --- | --- |
|  |  | 1 | 2 | 3 | 4 | 5 | 6 | 7 |
| sun-drying |  | 0 min | 40 min | 80 min | 120 min | 160 min | 200 min | 240 min |
| shade-drying |  | 0 h | 4 h | 8 h | 12 h | 16 h | 20 h | 24 h |
| oven-drying | 45 °C | 0 min | 40 min | 80 min | 120 min | 160 min | 200 min | 240 min |
|  | 55 °C | 0 min | 20 min | 40 min | 60 min | 80 min | 100 min | 120 min |
|  | 65 °C | 0 min | 20 min | 40 min | 60 min | 80 min | 100 min | 120 min |
|  | 75 °C | 0 min | 15 min | 30 min | 45 min | 60 min | 75 min | 90 min |
|  | 85 °C | 0 min | 12 min | 24 min | 36 min | 48 min | 60 min | 72 min |
|  | 95 °C | 0 min | 10 min | 20 min | 30 min | 40 min | 50 min | 60 min |
|  | 105 °C | 0 min | 8 min | 16 min | 24 min | 32 min | 40 min | 48 min |

**Table S2.** The results of precision and repeatability tests.

| Analytes | Precision test | | | | | | | Repeatability test | | | | | |
| --- | --- | --- | --- | --- | --- | --- | --- | --- | --- | --- | --- | --- | --- |
|  | Spiked  (μg/mL) | Intra-day (n=6) | | | Inter-day (n=9) | | | Contents (μg/mL, n=5) | | | | | RSD (%) |
|  |  | Found  (μg/mL) | RSD (%) | Precision (%) | Found  (μg/mL) | RSD (%) | Precision (%) |  |  |  |  |  |  |
| Neoastilbin | 63.20 | 64.79 | 1.47 | 102.51 | 66.32 | 2.01 | 104.94 | 48.66 | 47.25 | 49.38 | 44.46 | 46.65 | 3.62 |
| Astilbin | 239.60 | 238.65 | 1.18 | 99.60 | 244.30 | 1.83 | 101.96 | 336.29 | 328.20 | 340.96 | 307.15 | 323.14 | 3.59 |
| Neoisoastilbin | 56.50 | 56.97 | 1.17 | 100.84 | 58.42 | 1.84 | 103.40 | 10.32 | 10.09 | 10.50 | 9.47 | 9.97 | 3.50 |
| Isoastilbin | 87.50 | 88.40 | 1.16 | 101.03 | 90.64 | 1.84 | 103.59 | 75.46 | 73.74 | 76.65 | 69.00 | 72.44 | 3.61 |

**Table S3.** The results of linear ranges, LODs, LOQs and recovery tests.

| Analytes | Linear ranges  (μg/mL) | LOD  (μg/mL) | LOQ  (μg/mL) | Recovery test | | | |
| --- | --- | --- | --- | --- | --- | --- | --- |
|  |  |  |  | Spiked (μg/mL) | Found (μg/mL) | RSD (%) | Recovery (%) |
| Neoastilbin | 0.56-112.52 | 0.11 | 0.28 | 21.12 | 21.63 | 2.51 | 102.40 |
| Astilbin | 0.57-113.68 | 0.11 | 0.28 | 149.44 | 149.44 | 1.60 | 100.00 |
| Neoisoastilbin | 0.40-82.40 | 0.08 | 0.40 | 4.40 | 4.63 | 2.77 | 105.24 |
| Isoastilbin | 0.40-40.00 | 0.10 | 0.40 | 27.23 | 27.79 | 1.75 | 102.05 |

**Table S4.** Content of moisture and analytes in SGR during sun-drying.

| Samples | Analytes | Drying time (min) | | | | | | |
| --- | --- | --- | --- | --- | --- | --- | --- | --- |
|  |  | 0 | 40 | 80 | 120 | 160 | 200 | 240 |
| un-pretreated | Moisture (%) | 56.49±1.66 | 54.72±0.69 | 48.07±0.75 | 33.13±0.4 | 21.42±0.96 | 13.79±0.11 | 12.91±0.24 |
|  | Neo- (μg/g) | 416.5±6.96 | 461.85±22.98 | 370.57±9.30 | 551.89±17.45 | 572.09±4.89 | 394.26±16.85 | 461.85±31.10 |
|  | Astilbin (μg/g) | 3368.36±35.51 | 3810.32±126.19 | 3500.38±53.15 | 4723.61±162.15 | 5108.82±56.92 | 4169.07±124.09 | 4898.25±244.54 |
|  | Neoiso- (μg/g) | 125.05±1.55 | 146.11±3.81 | 156.10±5.31 | 220.79±8.97 | 254.53±5.90 | 184.65±5.23 | 204.75±14.01 |
|  | Iso- (μg/g) | 518.45±7.18 | 616.14±69.12 | 603.07±30.58 | 940.14±33.94 | 950.59±13.43 | 753.45±27.63 | 877.85±48.49 |
| boiled | Moisture (%) | 55.14±1.61 | 48.24±0.35 | 41.66±0.19 | 31.39±0.77 | 20.55±0.61 | 13.64±0.63 | 12.28±0.27 |
|  | Neo- (μg/g) | 1549.23±37.85 | 1657.54±754.94 | 1531.83±52.22 | 1656.25±49.00 | 1624.34±26.11 | 1466.07±43.84 | 1325.52±55.12 |
|  | Astilbin (μg/g) | 3386.11±174.17 | 3613.44±148.23 | 3470.14±68.69 | 3477.37±112.08 | 3572.69±48.32 | 3058.30±103.86 | 3141.35±96.40 |
|  | Neoiso- (μg/g) | 1517.53±36.45 | 1692.13±77.91 | 1644.74±40.57 | 1763.21±54.93 | 1699.31±24.05 | 1495.39±45.59 | 1495.75±51.38 |
|  | Iso- (μg/g) | 640.24±13.12 | 788.16±122.54 | 642.59±21.76 | 665.62±18.56 | 680.02±9.92 | 583.72±3.84 | 577.32±23.53 |
| steamed | Moisture (%) | 53.15±1.8 | 48.02±0.51 | 35.12±0.94 | 25.77±0.43 | 15.18±0.42 | 12.25±0.22 | 9.63±0.01 |
|  | Neo- (μg/g) | 3125.3±29.42 | 3290.04±134.13 | 2770.93±61.71 | 3299.74±38.29 | 2604.82±97.78 | 2561.92±125.29 | 2709.90±77.17 |
|  | Astilbin (μg/g) | 1437.91±14.52 | 1559.66±45.76 | 1288.53±47.86 | 1528.47±31.74 | 1178.70±44.29 | 1167.21±67.62 | 1250.79±16.43 |
|  | Neoiso- (μg/g) | 845.22±16.88 | 895.20±32.69 | 733.93±13.52 | 891.54±14.22 | 700.42±30.27 | 822.96±302.81 | 730.82±22.36 |
|  | Iso- (μg/g) | 938.22±10.29 | 974.82±34.96 | 817.60±13.62 | 991.81±14.50 | 781.23±34.40 | 766.60±33.90 | 804.91±26.07 |

**Table S5.** Content of moisture and analytes in SGR during shade-drying.

| Samples | Analytes | Drying time (h) | | | | | | |
| --- | --- | --- | --- | --- | --- | --- | --- | --- |
|  |  | 0 | 4 | 8 | 12 | 16 | 20 | 24 |
| un-pretreated | Moisture (%) | 56.49±1.66 | 51.98±1.53 | 37.08±0.55 | 26.14±1.12 | 19.04±0.73 | 13.15±0.32 | 14.13±0.21 |
|  | Neo- (μg/g) | 416.5±6.96 | 540.93±19.13 | 490.81±19.26 | 715.00±27.47 | 683.41±31.88 | 447.88±12.74 | 368.8±13.24 |
|  | Astilbin (μg/g) | 3368.36±35.51 | 3426.20±46.55 | 3623.14±109.92 | 4551.00±210.85 | 3824.34±159.60 | 3369.89±92.46 | 3482.74±186.86 |
|  | Neoiso- (μg/g) | 125.05±1.55 | 151.14±5.45 | 179.26±12.81 | 237.42±21.36 | 197.81±12.22 | 147.79±4.12 | 160.95±7.67 |
|  | Iso- (μg/g) | 518.45±7.18 | 625.10±16.38 | 722.41±31.68 | 959.01±47.13 | 794.24±33.94 | 641.95±19.20 | 656.45±33.68 |
| boiled | Moisture (%) | 55.14±1.61 | 52.47±0.65 | 34.64±0.52 | 24.96±0.36 | 15.58±0.31 | 16.83±0.17 | 13.61±0.22 |
|  | Neo- (μg/g) | 1549.23±37.85 | 1507.65±9.99 | 1302.64±0.32 | 1566.96±73.17 | 1294.90±28.04 | 1591.46±62.86 | 1364.64±61.8 |
|  | Astilbin (μg/g) | 3386.11±174.17 | 3115.49±97.29 | 3096.10±8.22 | 3513.20±140.67 | 2937.68±68.04 | 3010.64±115.37 | 3152.96±139.94 |
|  | Neoiso- (μg/g) | 1517.53±36.45 | 1502.93±46.67 | 1453.39±0.00 | 1674.89±80.78 | 1360.40±26.93 | 1468.46±45.95 | 1485.46±52.85 |
|  | Iso- (μg/g) | 640.24±13.12 | 604.83±7.68 | 581.16±1.28 | 671.06±29.76 | 557.16±11.84 | 632.35±20.48 | 598.43±27.19 |
| steamed | Moisture (%) | 53.15±1.8 | 38.66±0.54 | 27±0.99 | 17.24±0.47 | 16.06±0.79 | 13.42±0.43 | 12.65±1.02 |
|  | Neo- (μg/g) | 3125.3±29.42 | 2490.70±98.45 | 3137.12±61.87 | 2750.09±101.69 | 3054.60±138.97 | 3442.92±32.84 | 2851.09±39.36 |
|  | Astilbin (μg/g) | 1437.91±14.52 | 1130.05±39.00 | 1481.96±25.80 | 1339.09±42.82 | 1359.69±70.61 | 1502.77±38.56 | 1324.19±32.62 |
|  | Neoiso- (μg/g) | 845.22±16.88 | 682.47±22.75 | 854.74±14.58 | 765.52±29.94 | 797.59±28.34 | 880.17±2.44 | 779.17±6.09 |
|  | Iso- (μg/g) | 938.22±10.29 | 751.37±27.03 | 918.70±16.34 | 809.81±35.17 | 894.92±33.08 | 1002.42±4.97 | 853.54±5.29 |

**Table S6.** Content of moisture and analytes in SGR during oven-drying at 45°C.

| Samples | Analytes | Drying time (min) | | | | | | |
| --- | --- | --- | --- | --- | --- | --- | --- | --- |
|  |  | 0 | 40 | 80 | 120 | 160 | 200 | 240 |
| un-pretreated | Moisture (%) | 56.49±1.66 | 53.52±1.67 | 38.38±1.9 | 29.39±1.07 | 16.84±1.08 | 7.21±0.67 | 5.92±0.68 |
|  | Neo- (μg/g) | 416.5±6.96 | 588.80±17.49 | 445.30±19.04 | 513.08±25.43 | 531.90±21.92 | 695.82±39.74 | 468.72±23.01 |
|  | Astilbin (μg/g) | 3368.36±35.51 | 3868.00±134.66 | 3905.64±161.58 | 3970.98±154.04 | 4686.09±186.72 | 4970.72±211.51 | 5051.41±240.23 |
|  | Neoiso- (μg/g) | 125.05±1.55 | 162.21±9.78 | 181.77±13.52 | 201.45±11.89 | 240.29±16.72 | 278.53±27.18 | 224.61±11 |
|  | Iso- (μg/g) | 518.45±7.18 | 714.58±26.79 | 795.74±28.45 | 799.62±24.25 | 922.06±51.75 | 1065.40±38.23 | 1008.39±54.54 |
| boiled | Moisture (%) | 55.14±1.61 | 45.76±0.98 | 33.04±1.06 | 27.54±1.67 | 13.85±0.52 | 5.55±0.29 | 4.49±0.29 |
|  | Neo- (μg/g) | 1549.23±37.85 | 1388.28±34.69 | 1683.76±51.06 | 1521.51±87.03 | 1780.36±26.11 | 1451.89±53.13 | 1552.03±28.84 |
|  | Astilbin (μg/g) | 3386.11±174.17 | 3697.91±108.93 | 3729.03±99.48 | 2985.34±145.93 | 3838.92±71.98 | 2968.68±73.27 | 3858.09±70.60 |
|  | Neoiso- (μg/g) | 1517.53±36.45 | 1624.51±57.82 | 1765.48±43.58 | 1499.34±94.06 | 1864.45±39.13 | 1402.41±44.08 | 1784.15±25.86 |
|  | Iso- (μg/g) | 640.24±13.12 | 651.12±33.93 | 711.06±20.82 | 591.08±45.75 | 739.53±13.76 | 601.21±18.77 | 731.96±12.26 |
| steamed | Moisture (%) | 53.15±1.8 | 45.73±0.39 | 34.19±0.86 | 24.12±1.73 | 8.42±0.94 | 6.25±0.1 | 4.47±0.32 |
|  | Neo- (μg/g) | 3125.3±29.42 | 3121.52±132.78 | 2888.05±41.66 | 2511.55±66.68 | 3236.19±121.04 | 2903.52±128.23 | 2997.86±97.73 |
|  | Astilbin (μg/g) | 1437.91±14.52 | 1514.56±59.83 | 1346.32±13.80 | 1180.23±31.48 | 1483.05±31.04 | 1323.75±60.06 | 1348.29±37.04 |
|  | Neoiso- (μg/g) | 845.22±16.88 | 747.57±327.76 | 788.02±5.42 | 720.29±18.79 | 871.07±32.29 | 793.29±34.00 | 786.11±21.25 |
|  | Iso- (μg/g) | 938.22±10.29 | 944.36±37.38 | 860.36±8.51 | 760.54±18.85 | 955.92±38.07 | 859.72±36.36 | 886.17±26.67 |

**Table S7.** Content of moisture and analytes in SGR during oven-drying at 55°C.

| Samples | Analytes | Drying time (min) | | | | | | |
| --- | --- | --- | --- | --- | --- | --- | --- | --- |
|  |  | 0 | 20 | 40 | 60 | 80 | 100 | 120 |
| un-pretreated | Moisture (%) | 56.49±1.66 | 51.85±3.15 | 51.25±1.93 | 32.44±1.85 | 25.11±1.83 | 11.48±0.59 | 7.38±0.64 |
|  | Neo- (μg/g) | 416.5±6.96 | 501.82±30.46 | 375.73±11.55 | 373.31±15.06 | 437.35±11.83 | 441.43±4.54 | 578.11±12.24 |
|  | Astilbin (μg/g) | 3368.36±35.51 | 3840.56±127.54 | 3532.26±157.54 | 4439.19±172.12 | 4521.80±82.66 | 4291.29±123.59 | 4711.56±124.6 |
|  | Neoiso- (μg/g) | 125.05±1.55 | 155.92±9.16 | 138.87±5.24 | 184.41±7.92 | 194.46±5.78 | 193.50±3.44 | 255.25±5.54 |
|  | Iso- (μg/g) | 518.45±7.18 | 640.03±56.95 | 587.72±45.86 | 810.45±32.01 | 816.21±15.29 | 909.49±20.42 | 1070.25±27.55 |
| boiled | Moisture (%) | 55.14±1.61 | 47.62±1.99 | 42.18±0.39 | 34.53±4.19 | 19.88±0.22 | 10.84±0.81 | 5.86±0.54 |
|  | Neo- (μg/g) | 1549.23±37.85 | 1589.21±51.17 | 1616.93±58.67 | 1565.03±14.51 | 1546.98±40.94 | 1132.76±27.64 | 1607.9±61.31 |
|  | Astilbin (μg/g) | 3386.11±174.17 | 2937.13±105.90 | 3145.07±134.76 | 3269.32±43.39 | 3396.51±70.01 | 2555.64±46.10 | 3507.28±120.16 |
|  | Neoiso- (μg/g) | 1517.53±36.45 | 1431.84±44.85 | 1552.47±59.60 | 1567.19±16.16 | 1597.71±38.06 | 1269.33±14.85 | 1693.92±57.31 |
|  | Iso- (μg/g) | 640.24±13.12 | 616.78±18.59 | 627.23±24.32 | 646.43±9.60 | 630.11±31.67 | 461.07±10.38 | 686.1±21.07 |
| steamed | Moisture (%) | 53.15±1.8 | 46.29±0.97 | 37.48±0.19 | 30.66±1.1 | 17.11±0.77 | 8.41±0.43 | 6.61±0.5 |
|  | Neo- (μg/g) | 3125.3±29.42 | 2981.75±129.38 | 3123.58±152.60 | 2934.04±53.20 | 3066.85±82.53 | 2905.46±67.74 | 3141.42±128.57 |
|  | Astilbin (μg/g) | 1437.91±14.52 | 1350.05±46.86 | 1430.68±73.34 | 1311.92±32.27 | 1380.72±39.18 | 1330.98±41.24 | 1390.15±50.61 |
|  | Neoiso- (μg/g) | 845.22±16.88 | 802.86±39.98 | 846.18±28.39 | 775.58±14.32 | 801.42±23.43 | 783.23±11.22 | 813.63±27.52 |
|  | Iso- (μg/g) | 938.22±10.29 | 884.04±43.88 | 928.19±34.41 | 874.23±14.95 | 884.89±24.67 | 849.06±12.63 | 923.93±33.89 |

**Table S8.** Content of moisture and analytes in SGR during oven-drying at 65°C.

| Samples | Analytes | Drying time (min) | | | | | | |
| --- | --- | --- | --- | --- | --- | --- | --- | --- |
|  |  | 0 | 20 | 40 | 60 | 80 | 100 | 120 |
| un-pretreated | Moisture (%) | 56.49±1.66 | 48.35±0.73 | 43.43±2.81 | 23.46±0.44 | 17.52±0.62 | 7.34±0.77 | 7.36±0.73 |
|  | Neo- (μg/g) | 416.5±6.96 | 450.16±21.41 | 589.28±34.31 | 568.22±13.70 | 591.86±15.72 | 722.30±6.15 | 385.99±12.81 |
|  | Astilbin (μg/g) | 3368.36±35.51 | 3219.09±139.98 | 4371.27±175.08 | 4254.91±147.66 | 5449.77±103.14 | 4526.40±161.83 | 4219.86±131.40 |
|  | Neoiso- (μg/g) | 125.05±1.55 | 134.81±14.51 | 207.14±12.22 | 220.07±4.92 | 285.65±5.78 | 236.58±3.33 | 216.96±10.60 |
|  | Iso- (μg/g) | 518.45±7.18 | 540.84±47.37 | 838.61±36.20 | 875.30±26.93 | 1051.91±16.11 | 974.91±18.82 | 829.86±29.11 |
| boiled | Moisture (%) | 55.14±1.61 | 45.74±1.65 | 39.6±0.29 | 19.6±0.94 | 12.62±0.19 | 7.94±0.36 | 6.91±0.64 |
|  | Neo- (μg/g) | 1549.23±37.85 | 1486.48±47.74 | 1610.48±79.30 | 1293.93±1.93 | 1437.38±21.60 | 1540.21±45.77 | 1534.62±70.6 |
|  | Astilbin (μg/g) | 3386.11±174.17 | 3154.72±69.24 | 3275.56±152.84 | 3050.41±2.63 | 3188.79±9.53 | 3379.42±88.41 | 3610.05±146.90 |
|  | Neoiso- (μg/g) | 1517.53±36.45 | 1495.03±44.27 | 1621.40±73.96 | 1402.41±0.72 | 1478.52±3.59 | 1654.79±37.70 | 1726.95±65.83 |
|  | Iso- (μg/g) | 640.24±13.12 | 613.37±16.69 | 645.79±30.07 | 573.48±0.64 | 605.47±6.40 | 643.55±16.32 | 674.16±30.16 |
| steamed | Moisture (%) | 53.15±1.8 | 47.36±0.53 | 40.04±0.16 | 21.46±3 | 12.61±1.48 | 7.82±0.78 | 4.95±0.63 |
|  | Neo- (μg/g) | 3125.3±29.42 | 3479.32±127.10 | 2886.33±49.05 | 2761.10±82.92 | 3148.08±40.38 | 3054.60±112.40 | 2854.96±64.03 |
|  | Astilbin (μg/g) | 1437.91±14.52 | 1619.48±54.61 | 1310.39±27.58 | 1247.94±26.09 | 1368.02±37.33 | 1387.74±56.01 | 1319.15±22.6 |
|  | Neoiso- (μg/g) | 845.22±16.88 | 942.16±31.25 | 790.17±9.48 | 755.83±24.10 | 805.25±11.07 | 822.49±20.90 | 763.13±11.14 |
|  | Iso- (μg/g) | 938.22±10.29 | 1022.56±36.38 | 858.87±11.97 | 827.84±27.45 | 913.05±12.05 | 907.08±25.54 | 842.02±14.64 |

**Table S9.** Content of moisture and analytes in SGR during oven-drying at 75°C.

| Samples | Analytes | Drying time (min) | | | | | | |
| --- | --- | --- | --- | --- | --- | --- | --- | --- |
|  |  | 0 | 15 | 30 | 45 | 60 | 75 | 90 |
| un-pretreated | Moisture (%) | 56.49±1.66 | 47.78±0.97 | 34.44±2.48 | 27.49±0.26 | 14.41±0.96 | 8.93±0.36 | 6.69±0.51 |
|  | Neo- (μg/g) | 416.5±6.96 | 321.73±14.13 | 446.48±21.41 | 486.13±25.65 | 659.12±14.55 | 513.64±16.14 | 410.92±9.36 |
|  | Astilbin (μg/g) | 3368.36±35.51 | 3516.26±171.57 | 4066.69±124.41 | 4051.90±196.82 | 4389.23±86.60 | 5009.78±169.39 | 5264.62±97.45 |
|  | Neoiso- (μg/g) | 125.05±1.55 | 125.53±5.69 | 178.90±13.26 | 198.47±15.51 | 228.20±6.27 | 221.50±6.59 | 217.91±2.71 |
|  | Iso- (μg/g) | 518.45±7.18 | 561.96±26.42 | 710.58±22.34 | 813.76±41.44 | 938.64±20.05 | 1031.43±31.10 | 993.46±13.45 |
| boiled | Moisture (%) | 55.14±1.61 | 44.55±1.75 | 42.79±0.5 | 23.62±1.78 | 21.17±0.33 | 5.61±0.46 | 5.98±0.14 |
|  | Neo- (μg/g) | 1549.23±37.85 | 1296.84±24.18 | 1357.44±38.36 | 1190.78±0.00 | 1546.01±29.01 | 1464.78±40.62 | 1404.18±46.97 |
|  | Astilbin (μg/g) | 3386.11±174.17 | 3130.94±102.22 | 2878.52±31.88 | 2968.90±4.60 | 3595.69±47.66 | 3143.43±93.02 | 3434.31±148.75 |
|  | Neoiso- (μg/g) | 1517.53±36.45 | 1409.59±43.80 | 1361.12±6.82 | 1339.22±2.15 | 1658.38±17.59 | 1570.78±47.75 | 1654.67±54.24 |
|  | Iso- (μg/g) | 640.24±13.12 | 558.44±4.16 | 571.56±12.16 | 554.28±1.92 | 690.90±11.20 | 620.51±18.24 | 633.63±26.71 |
| steamed | Moisture (%) | 53.15±1.8 | 42.88±1.62 | 42.51±0.09 | 21.34±1.59 | 14.29±0.29 | 6.01±0.56 | 3.39±0.4 |
|  | Neo- (μg/g) | 3125.3±29.42 | 3095.43±145.92 | 3251.44±126.27 | 2742.14±53.54 | 3273.15±144.69 | 2805.75±105.33 | 2868.28±73.23 |
|  | Astilbin (μg/g) | 1437.91±14.52 | 1418.85±78.30 | 1513.29±55.05 | 1237.20±28.32 | 1525.78±73.16 | 1258.67±39.89 | 1310.61±54.64 |
|  | Neoiso- (μg/g) | 845.22±16.88 | 850.49±34.19 | 903.86±32.60 | 757.86±11.46 | 901.71±37.92 | 769.11±23.47 | 773.42±13.52 |
|  | Iso- (μg/g) | 938.22±10.29 | 916.46±37.63 | 973.41±36.16 | 831.57±13.85 | 954.64±24.29 | 843.94±27.36 | 850.77±14.73 |

**Table S10.** Content of moisture and analytes in SGR during oven-drying at 85°C.

| Samples | Analytes | Drying time (min) | | | | | | |
| --- | --- | --- | --- | --- | --- | --- | --- | --- |
|  |  | 0 | 12 | 24 | 36 | 48 | 60 | 72 |
| un-pretreated | Moisture (%) | 56.49±1.66 | 49.47±2.24 | 46.49±2.02 | 29.51±0.94 | 18.19±0.35 | 5.01±0.53 | 2.95±0.68 |
|  | Neo- (μg/g) | 416.5±6.96 | 340.43±14.77 | 522.66±19.95 | 484.84±17.47 | 491.77±13.29 | 495.16±15.55 | 543.08±15.94 |
|  | Astilbin (μg/g) | 3368.36±35.51 | 2971.75±58.07 | 3857.65±141.60 | 4351.98±219.05 | 3616.73±112.03 | 4402.16±187.22 | 5190.99±168.56 |
|  | Neoiso- (μg/g) | 125.05±1.55 | 120.50±3.53 | 188.71±9.16 | 193.02±8.97 | 195.42±8.98 | 210.97±9.13 | 227.01±5.95 |
|  | Iso- (μg/g) | 518.45±7.18 | 510.77±9.24 | 743.48±28.48 | 781.66±33.57 | 737.29±20.01 | 861.43±32.85 | 1009.25±28.57 |
| boiled | Moisture (%) | 55.14±1.61 | 44.44±1.58 | 36.52±0.56 | 26.29±0.6 | 19.14±1.54 | 3.97±0.24 | 4.79±0.09 |
|  | Neo- (μg/g) | 1549.23±37.85 | 1109.77±56.89 | 1177.89±7.09 | 1209.80±25.47 | 1433.83±31.59 | 1434.48±76.72 | 1554.39±80.92 |
|  | Astilbin (μg/g) | 3386.11±174.17 | 2822.75±131.69 | 2476.21±23.34 | 2582.38±19.72 | 2984.68±42.07 | 2934.39±89.73 | 3054.58±177.42 |
|  | Neoiso- (μg/g) | 1517.53±36.45 | 1294.70±53.83 | 1258.08±9.33 | 1279.27±29.08 | 1500.06±22.26 | 1422.87±65.70 | 1466.79±93.02 |
|  | Iso- (μg/g) | 640.24±13.12 | 504.16±20.06 | 480.37±4.16 | 513.65±11.20 | 595.24±10.24 | 596.20±29.76 | 632.99±34.89 |
| steamed | Moisture (%) | 53.15±1.8 | 45.84±2.66 | 34.67±1.67 | 21.6±0.79 | 13.28±0.6 | 3.22±0.2 | 3.2±0.23 |
|  | Neo- (μg/g) | 3125.3±29.42 | 2513.70±66.10 | 2974.87±23.16 | 2756.96±117.42 | 2992.28±7.92 | 2532.18±96.10 | 2926.73±94.87 |
|  | Astilbin (μg/g) | 1437.91±14.52 | 1118.00±18.76 | 1335.37±4.39 | 1176.50±29.90 | 1360.78±3.28 | 1137.94±52.55 | 1316.74±58.43 |
|  | Neoiso- (μg/g) | 845.22±16.88 | 669.79±22.44 | 790.89±13.74 | 701.86±26.80 | 788.74±2.95 | 686.78±24.38 | 785.87±14.65 |
|  | Iso- (μg/g) | 938.22±10.29 | 748.17±24.19 | 875.51±13.61 | 794.24±30.45 | 876.36±3.78 | 754.14±26.59 | 877.64±19.24 |

**Table S11.** Content of moisture and analytes in SGR during oven-drying at 95°C.

| Samples | Analytes | Drying time (min) | | | | | | |
| --- | --- | --- | --- | --- | --- | --- | --- | --- |
|  |  | 0 | 10 | 20 | 30 | 40 | 50 | 60 |
| un-pretreated | Moisture (%) | 56.49±1.66 | 45.33±0.95 | 41.81±0.67 | 26.9±1.00 | 20.46±0.48 | 4.67±0.48 | 4.48±0.77 |
|  | Neo- (μg/g) | 416.5±6.96 | 426.17±23.76 | 339.36±12.26 | 444.01±20.67 | 485.92±15.71 | 634.41±6.84 | 619.15±20.61 |
|  | Astilbin (μg/g) | 3368.36±35.51 | 2968.03±125.21 | 3189.34±70.49 | 3989.34±107.61 | 5474.10±146.18 | 4419.47±83.93 | 4557.74±149.98 |
|  | Neoiso- (μg/g) | 125.05±1.55 | 141.32±7.47 | 137.02±4.88 | 201.64±8.34 | 258.12±12.52 | 227.49±4.70 | 228.44±8.68 |
|  | Iso- (μg/g) | 518.45±7.18 | 569.85±26.07 | 564.52±14.15 | 810.45±31.70 | 1007.33±23.36 | 973.20±13.43 | 938.86±36.16 |
| boiled | Moisture (%) | 55.14±1.61 | 46.83±2.1 | 40.5±1.04 | 24.58±0.7 | 17.34±1.21 | 5.06±0.36 | 3.76±0.34 |
|  | Neo- (μg/g) | 1549.23±37.85 | 1682.90±294.20 | 1496.37±51.61 | 1335.97±22.33 | 1641.10±73.01 | 1451.89±21.92 | 1683.98±68.65 |
|  | Astilbin (μg/g) | 3386.11±174.17 | 3101.03±135.35 | 2982.27±75.14 | 3176.50±72.01 | 3586.33±158.96 | 3258.47±56.20 | 3437.6±149.69 |
|  | Neoiso- (μg/g) | 1517.53±36.45 | 1430.17±68.89 | 1445.97±50.54 | 1496.04±32.23 | 1760.16±57.37 | 1592.68±10.05 | 1696.08±64.78 |
|  | Iso- (μg/g) | 640.24±13.12 | 680.13±82.59 | 615.50±22.15 | 587.68±11.41 | 690.90±25.98 | 612.83±3.52 | 702.74±25.54 |
| steamed | Moisture (%) | 53.15±1.8 | 51.77±1.22 | 40.43±2.35 | 31.74±1.37 | 12.9±0.55 | 7.11±1.56 | 2.88±0.19 |
|  | Neo- (μg/g) | 3125.3±29.42 | 3200.30±123.02 | 3053.57±139.82 | 3155.60±30.60 | 2998.51±85.35 | 2655.96±86.57 | 2854.31±135.85 |
|  | Astilbin (μg/g) | 1437.91±14.52 | 1513.73±68.42 | 1365.47±60.83 | 1417.32±29.57 | 1349.17±45.03 | 1209.37±45.42 | 1273.79±58.85 |
|  | Neoiso- (μg/g) | 845.22±16.88 | 874.42±27.56 | 799.84±31.94 | 835.17±5.37 | 786.35±26.76 | 706.41±25.56 | 743.50±34.05 |
|  | Iso- (μg/g) | 938.22±10.29 | 950.80±31.38 | 891.89±37.41 | 930.97±5.02 | 875.30±28.07 | 786.56±26.71 | 848.21±39.46 |

**Table S12.** Content of moisture and analytes in SGR during oven-drying at 105°C.

| Samples | Analytes | Drying time (min) | | | | | | |
| --- | --- | --- | --- | --- | --- | --- | --- | --- |
|  |  | 0 | 8 | 16 | 24 | 32 | 40 | 48 |
| un-pretreated | Moisture (%) | 56.49±1.66 | 51.23±0.75 | 43.75±0.67 | 33.91±2.52 | 27.45±0.22 | 6.57±0.19 | 4.58±0.22 |
|  | Neo- (μg/g) | 416.5±6.96 | 424.03±8.84 | 440.14±14.85 | 486.99±16.67 | 887.35±32.73 | 450.42±26.24 | 551.68±22.07 |
|  | Astilbin (μg/g) | 3368.36±35.51 | 3203.14±76.65 | 4802.71±217.85 | 4395.37±136.61 | 4545.47±179.70 | 5005.92±177.80 | 4822.65±200.65 |
|  | Neoiso- (μg/g) | 125.05±1.55 | 120.26±18.69 | 216.96±10.70 | 214.32±6.99 | 288.04±16.45 | 215.81±14.11 | 236.34±14.52 |
|  | Iso- (μg/g) | 518.45±7.18 | 504.16±48.69 | 798.29±36.06 | 824.10±26.28 | 1023.75±48.73 | 884.98±27.75 | 978.53±33.33 |
| boiled | Moisture (%) | 55.14±1.61 | 52.15±0.3 | 37.66±2.22 | 29.28±1.09 | 22.56±1.05 | 8.38±0.16 | 1.78±0.25 |
|  | Neo- (μg/g) | 1549.23±37.85 | 1632.83±41.99 | 1983.44±52.54 | 1859.01±262.07 | 1590.49±30.30 | 1395.80±25.79 | 1784.12±29.5 |
|  | Astilbin (μg/g) | 3386.11±174.17 | 2999.80±154.59 | 3825.44±78.23 | 3168.74±0.00 | 3087.56±56.20 | 3248.61±59.49 | 3989.12±81.48 |
|  | Neoiso- (μg/g) | 1517.53±36.45 | 1466.31±73.35 | 1838.24±40.93 | 1570.42±21.54 | 1549.24±19.03 | 1545.65±19.75 | 1901.91±33.5 |
|  | Iso- (μg/g) | 640.24±13.12 | 611.23±17.66 | 783.04±16.32 | 708.82±55.35 | 616.67±11.20 | 613.47±10.56 | 776.54±17.49 |
| steamed | Moisture (%) | 53.15±1.8 | 48.26±0.34 | 44.34±1.31 | 29.84±1.28 | 20.17±0.71 | 5.09±0.53 | 4.93±0.41 |
|  | Neo- (μg/g) | 3125.3±29.42 | 3158.61±57.68 | 2794.57±44.52 | 2945.64±132.41 | 3207.07±68.54 | 2689.06±99.43 | 3182.03±122.51 |
|  | Astilbin (μg/g) | 1437.91±14.52 | 1469.91±12.41 | 1292.42±15.27 | 1350.92±58.07 | 1456.16±52.82 | 1213.54±24.81 | 1422.36±58.21 |
|  | Neoiso- (μg/g) | 845.22±16.88 | 866.76±15.94 | 767.92±11.96 | 797.36±31.19 | 861.74±17.33 | 727.95±24.83 | 843.55±23.2 |
|  | Iso- (μg/g) | 938.22±10.29 | 923.07±15.51 | 832.21±13.23 | 868.68±35.32 | 943.82±21.28 | 800.43±29.92 | 937.37±29.49 |

**Table S13.** Bioactive ingredient content and DPPH**·**, ABTS**·^+^**, XO and α-Glu(n=3) inhibitory effects of the 27 dried SGR.

| No. | Content (μg/g) | | | |  | IC_50_ (μg/mL) | IC_50_ (μg/mL) | | | IC_50_ (mg/mL) | | IC_50_ (μg/mL) |
| --- | --- | --- | --- | --- | --- | --- | --- | --- | --- | --- | --- | --- |
|  | Neoastilbin | Astibin | Neoisoastilbin | Isoastilbin |  | DPPH**·** | | ABTS**·^+^** | XO | | α-Glu | |
| S1 | 461.85±31.10 | 4898.25±244.54 | 204.75±14.01 | 877.85±48.49 |  | 346.05±2.92 | | 76.01±0.28 | 3.32±0.04 | | 243.79±7.83 | |
| S2 | 368.80±13.24 | 3482.74±186.86 | 160.95±7.67 | 656.45±33.68 |  | 556.55±22.83 | | 109.68±1.8 | 6.18±0.12 | | 324.31±27.32 | |
| S3 | 468.72±23.01 | 5051.41±240.23 | 224.61±11.00 | 1008.39±54.54 |  | 371.15±10.54 | | 71.52±0.95 | 7.04±0.08 | | 262.05±7.79 | |
| S4 | 578.11±12.24 | 4711.56±124.60 | 255.25±5.54 | 1070.25±27.55 |  | 336.68±9.44 | | 71.22±1.19 | 4.43±0.13 | | 248.42±32.59 | |
| S5 | 385.99±12.81 | 4219.86±131.40 | 216.96±10.60 | 829.86±29.11 |  | 400.1±11.28 | | 83.84±1.44 | 5.48±0.10 | | 459.09±9.93 | |
| S6 | 410.92±9.36 | 5264.62±97.45 | 217.91±2.71 | 993.46±13.45 |  | 318.58±9.61 | | 74.41±1.24 | 5.59±0.13 | | 461.39±8.31 | |
| S7 | 543.08±15.94 | 5190.99±168.56 | 227.01±5.95 | 1009.25±28.57 |  | 340.58±9.8 | | 70.57±1.32 | 5.69±0.06 | | 330.16±27.09 | |
| S8 | 619.15±20.61 | 4557.74±149.98 | 228.44±8.68 | 938.86±36.16 |  | 335.00±6.52 | | 71.66±3.33 | 5.95±0.37 | | 276.67±4.64 | |
| S9 | 551.68±22.07 | 4822.65±200.65 | 236.34±14.52 | 978.53±33.33 |  | 348.38±10.26 | | 71.46±0.82 | 4.45±0.09 | | 281.58±13.23 | |
| S10 | 1325.52±55.12 | 3141.35±96.40 | 1495.75±51.38 | 577.32±23.53 |  | 375.03±4.86 | | 66.29±0.52 | 5.64±0.24 | | 377.96±8.40 | |
| S11 | 1364.64±61.80 | 3152.96±139.94 | 1485.46±52.85 | 598.43±27.19 |  | 353.53±7.02 | | 67.5±1.14 | 4.67±0.12 | | 323.77±6.10 | |
| S12 | 1552.03±28.84 | 3858.09±70.60 | 1784.15±25.86 | 731.96±12.26 |  | 296.87±1.82 | | 69.68±0.33 | 4.99±0.07 | | 264.00±8.31 | |
| S13 | 1607.90±61.31 | 3507.28±120.16 | 1693.92±57.31 | 686.10±21.07 |  | 306.93±8.19 | | 66.78±1.53 | 4.50±0.07 | | 251.66±3.27 | |
| S14 | 1534.62±70.60 | 3610.05±146.90 | 1726.95±65.83 | 674.16±30.16 |  | 295.75±7.83 | | 73.45±1.25 | 3.47±0.09 | | 235.25±8.29 | |
| S15 | 1404.18±46.97 | 3434.31±148.75 | 1654.67±54.24 | 633.63±26.71 |  | 331.63±13.94 | | 77.88±1.48 | 4.71±0.06 | | 329.4±9.48 | |
| S16 | 1554.39±80.92 | 3054.58±177.42 | 1466.79±93.02 | 632.99±34.89 |  | 322.45±5.82 | | 75.77±0.56 | 4.00±0.11 | | 276.51±5.81 | |
| S17 | 1683.98±68.65 | 3437.60±149.69 | 1696.08±64.78 | 702.74±25.54 |  | 282.48±9.51 | | 66.79±1.15 | 3.94±0.05 | | 377.33±4.74 | |
| S18 | 1784.12±29.50 | 3989.12±81.48 | 1901.91±33.50 | 776.54±17.49 |  | 268.17±2.11 | | 61.68±0.73 | 4.54±0.19 | | 288±1.41 | |
| S19 | 2709.90±77.17 | 1250.79±16.43 | 730.82±22.36 | 804.91±26.07 |  | 356.93±5.05 | | 78.6±1.43 | 6.58±0.29 | | 1294.17±14.10 | |
| S20 | 2851.09±39.36 | 1324.19±32.62 | 779.17±6.09 | 853.54±5.29 |  | 323.88±14.22 | | 72.93±0.56 | 6.16±0.20 | | 796.70±48.11 | |
| S21 | 2997.86±97.73 | 1348.29±37.04 | 786.11±21.25 | 886.17±26.67 |  | 319.85±12.68 | | 72.97±0.89 | 5.44±0.17 | | 1049.11±22.59 | |
| S22 | 3141.42±128.57 | 1390.15±50.61 | 813.63±27.52 | 923.93±33.89 |  | 305.90±7.68 | | 65.78±0.97 | 6.85±0.17 | | 1519.51±26.77 | |
| S23 | 2854.96±64.03 | 1319.15±22.60 | 763.13±11.14 | 842.02±14.64 |  | 314.33±2.88 | | 73.5±0.94 | 7.11±0.29 | | 1091.06±30.17 | |
| S24 | 2868.28±73.23 | 1310.61±54.64 | 773.42±13.52 | 850.77±14.73 |  | 314.05±4.56 | | 71.58±0.41 | 7.09±0.09 | | 993.89±22.10 | |
| S25 | 2926.73±94.87 | 1316.74±58.43 | 785.87±14.65 | 877.64±19.24 |  | 321.73±12.38 | | 68.63±0.25 | 7.2±0.33 | | 936.48±28.18 | |
| S26 | 2854.31±135.85 | 1273.79±58.85 | 743.50±34.05 | 848.21±39.46 |  | 339.45±8.42 | | 68.29±0.83 | 7.55±0.41 | | 739.44±12.99 | |
| S27 | 3182.03±122.51 | 1422.36±58.21 | 843.55±23.20 | 937.37±29.49 |  | 283.8±12.11 | | 58.63±0.81 | 5.83±0.25 | | 949.07±30.68 | |
| P |  |  |  |  |  | 3.32±0.03 | | 2.15±0.03 | 0.02±0.00 | | 398.20±21.63 | |

*The positive controls (P) in DPPH**·** and ABTS**·^+^** are both ascorbic acid, and allopurinol and acarbose are the positive controls for XO and α-Glu respectively.

**S14. The operation steps and equations of entropy weight and TOPSIS analysis:**

First, a normalized decision matrix was established, and if there existed *m* alternatives (*i* = 1, 2,...m) and *n* criteria (*j* = 1, 2,...n), then a multi-objective decision matrix *X* = (*X_ij_*) _n × m_ was established (1):

$X=\left[ X_{ij} \right]=\left[ \begin{matrix} X_{11} & X_{12} & \cdots& X_{1n} \\ X_{21} & X_{22} & \cdots& X_{2n} \\ & & & \vdots\\ X_{m1} & X_{m2} & & X_{mn} \end{matrix} \right]$ （1）

Among the five evaluation indicators (the IC_50_ values, and the production of NO in RAW264.7 cells with SGR at concentrations of 0.53 mg/mL), the bioactive data were standardized by Equation (2).

$\text{X'}_{\text{ij}}\text{=(}\text{X}_{\text{max}}-\text{X}_{\text{ij}}\text{)/(}\text{X}_{\text{max}}-\text{X}_{\text{min}}\text{)}$ （2）

Subsequently, the decision matrix should be normalized. The normalization procedure is shown in Equation (3).

$\text{r}_{\text{ij}}\text{=}\text{X'}_{\text{ij}}\text{/}\sum_{\text{i=1}}^{\text{m}} \text{X'}_{\text{ij}}$ （3）

The entropy value *e_j_* and weight vectors *w_j_* can be computed using the expressions given in Equation (4) and (5), respectively.

$\text{e}_{\text{j}}\text{=}-\text{1/}\ln\text{m}\sum_{\text{i=1}}^{\text{m}} \text{r}_{\text{ij}}\ln\text{r}_{\text{ij}}\text{,}\text{i}\text{=1,2}\text{⋯⋯}\text{m and j=1,2}\text{⋯⋯}\text{n}$ （4）

$\text{w}_{\text{j}}\text{=(1}{-\text{e}}_{\text{j}}\text{)/}\sum_{\text{j=1}}^{\text{n}} \text{ (1}-\text{e}_{\text{j}}\text{)}\text{, j=1,2}\text{⋯⋯}\text{n}$ （5）

The TOPSIS method was then carried out. The same direction matrix established in Equations (2) was normalized using Equation (6).

${\bar{\text{X}}}_{\text{ij}}\text{=}{\text{X}^{\text{'}}}_{\text{ij}}\text{/}{\text{(}\sum_{\text{i=1}}^{\text{m}} {\text{X}^{\text{'}}}_{\text{ij}}^{\text{2}}\text{)}}^{\text{1/2}}$ （6）

Then, Equation (7) was used to weight the normalized data to construct a weighted decision matrix. According to Equations (8) and (9), the optimal and worst vector values of each evaluation index were obtained.

$\text{V}_{\text{ij}}\text{=}\text{w}_{\text{j}}\text{×}{\bar{\text{X}}}_{\text{ij}}$ （7）

$\text{V}_{\text{j}}^{\text{+}}\text{=max(}\text{V}_{\text{1j}}\text{,}\text{V}_{\text{2j}}\text{,}\text{⋯}\text{,}\text{V}_{\text{nj}}\text{) }$ （8）

$\text{V}_{\text{j}}^{-}\text{=min(}\text{V}_{\text{1j}}\text{,}\text{V}_{\text{2j}}\text{,}\text{⋯}\text{,}\text{V}_{\text{nj}}\text{) }$ （9）

The distances *S_i_^+^* between each evaluation index and the positive ideal solution, as well as distances *S_i_^–^* between each evaluation index and negative ideal solution, were calculated using Equations (10)-(12), and the closeness *P_i_* of the optimal solution was obtained and sorted. The sorting result is the optimal order combining the antioxidant, XO and α-Glu inhibitory, and anti-inflammatory activities.

$\text{S}_{\text{i}}^{\text{+}}\text{=}\left[ \sum_{\text{j}}^{\text{m}} {\text{ (}\text{V}_{\text{ij}}{-\text{V}}_{\text{j}}^{\text{+}}\text{) }}^{\text{2}} \right]^{\text{1/2}}$ （10）

$\text{S}_{\text{i}}^{-}\text{=}\left[ \sum_{\text{j}}^{\text{m}} {\text{ (}\text{V}_{\text{ij}}{-\text{V}}_{\text{j}}^{-}\text{) }}^{\text{2}} \right]^{\text{1/2}}$ （11）

$\text{P}_{\text{i}}\text{=}\text{S}_{\text{i}}^{-}\text{/(}\text{S}_{\text{i}}^{\text{+}}\text{+}\text{S}_{\text{i}}^{-}\text{)}$ （12）

**Table S15** Comprehensive evaluation result of entropy weight and TOPSIS model

| Treatment and drying methods | | S^+^ | S^-^ | P | Rank |
| --- | --- | --- | --- | --- | --- |
| Un-pretreated | Su.D. | 0.0185 | 0.1609 | 0.8967 | 2 |
|  | Sh.D. | 0.1074 | 0.0737 | 0.407 | 20 |
| Ov.D | 45℃ | 0.1308 | 0.0714 | 0.3533 | 21 |
|  | 55℃ | 0.0402 | 0.1286 | 0.7618 | 5 |
|  | 65℃ | 0.0831 | 0.0871 | 0.5119 | 16 |
|  | 75℃ | 0.0825 | 0.0883 | 0.5171 | 14 |
|  | 85℃ | 0.084 | 0.0908 | 0.5193 | 13 |
|  | 95℃ | 0.0931 | 0.0857 | 0.4791 | 17 |
|  | 105℃ | 0.0416 | 0.1265 | 0.7526 | 6 |
| Boiled | Su.D. | 0.0841 | 0.0884 | 0.5125 | 15 |
|  | Sh.D. | 0.0486 | 0.1198 | 0.7113 | 9 |
| Ov.D | 45℃ | 0.0594 | 0.112 | 0.6535 | 11 |
|  | 55℃ | 0.0428 | 0.1262 | 0.7469 | 8 |
|  | 65℃ | 0.0161 | 0.1571 | 0.907 | 1 |
|  | 75℃ | 0.0517 | 0.1166 | 0.6927 | 10 |
|  | 85℃ | 0.0312 | 0.1386 | 0.8162 | 4 |
|  | 95℃ | 0.0253 | 0.1414 | 0.8482 | 3 |
|  | 105℃ | 0.0427 | 0.1282 | 0.7503 | 7 |
| Steamed | Su.D. | 0.1233 | 0.0517 | 0.2954 | 22 |
|  | Sh.D. | 0.1027 | 0.0713 | 0.4098 | 19 |
| Ov.D | 45℃ | 0.082 | 0.0895 | 0.5219 | 12 |
|  | 55℃ | 0.1361 | 0.0434 | 0.2419 | 25 |
|  | 65℃ | 0.1432 | 0.0363 | 0.2024 | 27 |
|  | 75℃ | 0.1363 | 0.0479 | 0.26 | 23 |
|  | 85℃ | 0.14 | 0.0462 | 0.2484 | 24 |
|  | 95℃ | 0.1509 | 0.0475 | 0.2393 | 26 |
|  | 105℃ | 0.0933 | 0.079 | 0.4584 | 18 |


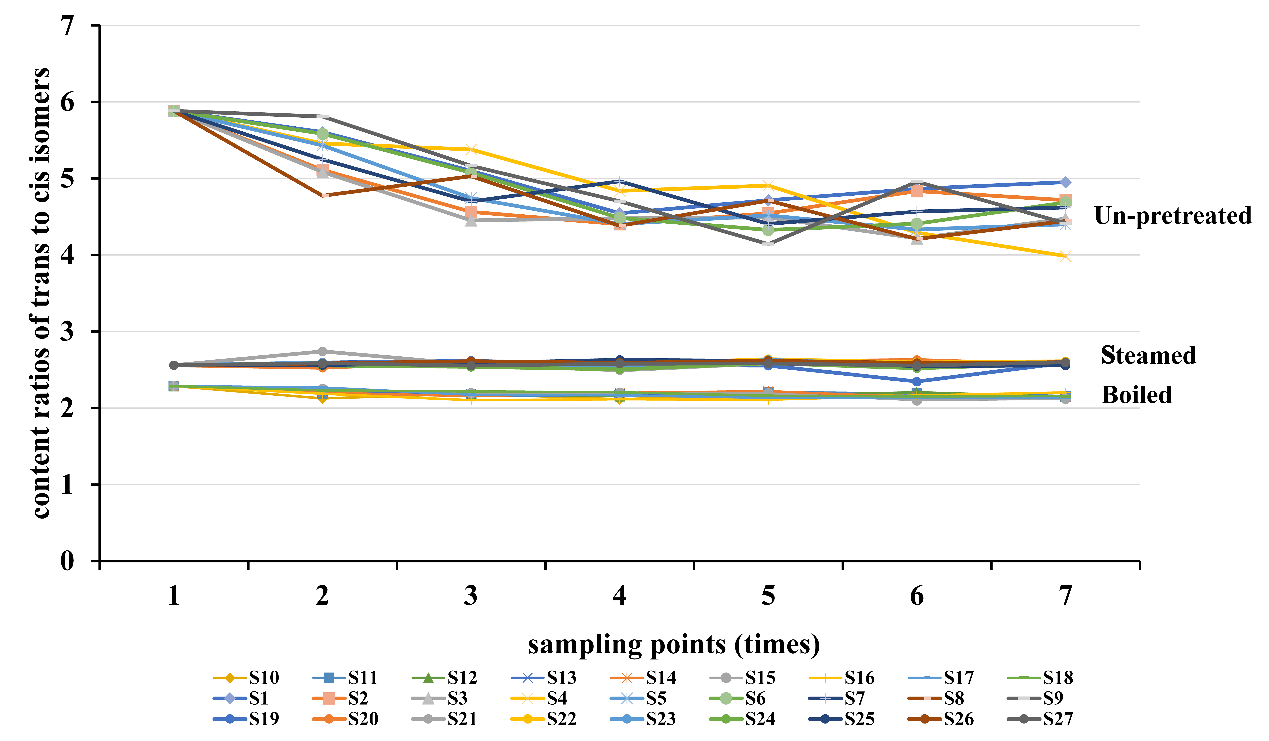


**Figure S1.** The content rations of trans to cis isomers of SGR throughout drying processing at different sampling times.
