# Supplementary material for: Influence of different pretreatments and drying methods on the chemical compositions and bioactivities of Smilacis Glabrae Rhizoma
Source: Chin Med. 2022 May 6;17:54. doi: 10.1186/s13020-022-00614-7 (PMC9074193; doi:10.1186/s13020-022-00614-7)
Supplement: Supplementary file 2 — Additional file 2: S1. Optimization of Extraction Processing for Bioactive Ingredients. Table S1. The Box-Behnken design experiment scheme. Table S2. ANOVA of quadratic model terms of three factor variables on responses. Table S3. Predicted and experimental values of the responses obtained under the optimal extraction conditions. Figure S1. Result of the single factor experiments. Figure S2. Response surface plots corresponding to the desirability function. [file 13020_2022_614_MOESM2_ESM.docx]

**Influence of different pretreatments and drying methods on the chemical compositions and bioactivities of Smilacis Glabrae Rhizoma**

Juanjuan Qiao^1^, Gengyu Lu^1^, Gang Wu^2^, Hui Liu^3^, Wanli Wang^1^, Tianmao Zhang^1^, Guoyong Xie^1,^* and Minjian Qin^1,^*

^*^Correspondence: guoyongxie321@163.com; minjianqin@163.com

^1^ Department of Resources Science of Traditional Chinese Medicines, School of Traditional Chinese Pharmacy, China Pharmaceutical University, Nanjing 211198, China; kfq6310@163.com (JJQ); njlugengyu@126.com (GYL); wanliw23@163.com (WLW)

^2^ The Teaching Experiments Center of Traditional Chinese Medicines, School of Traditional Chinese Pharmacy, China Pharmaceutical University, Nanjing 211198, China; woosmail@163.com (GW)

^3^ Yangzhou Center for Food and Drug Control, Yangzhou 225000, China; liu_huiabc@163.com (HL)

**S1.** **Optimization of Extraction Processing for Bioactive Ingredients**

The powders (0.3 g; 20 mesh) were placed in a 50 mL centrifuge tube and mixed with extraction solution. The extraction process was carried out using an ultrasonic cleaning bath (KH-300DB, Kunshan ultrasonic instrument Co., Ltd., Jiangsu, China). After ultrasonic extraction, the extracts were centrifuged at 10 000 g/min for 10 min. The supernatant was being collected in a 25 mL brown volumetric flask and diluted to volume with extraction solution. The samples were filtered through a 0.22 μm microfiltration membrane before UHPLC.

The optimization of extraction processing for the bioactive ingredients of SGR samples were carried out by response surface methodology (RSM), which is a widely used empirical modeling methodology. Five factors including extraction methods (thermal reflux, ultrasound, and cold soak), extraction solution (methanol and ethanol both at the concentration of 20%, 40%, 60%, 80%, 100%), extraction time (10, 20, 30, and 40 min), volume of extraction solvent (3, 6, 8, and 10 mL) and extraction times (1, 2, and 3 times) were used for the optimization of single factor conditions, and the total peak (peak area > 5%) area of SGR extracts was the evaluation index. The variables in RSM process were extraction time (*A*), ethanol concentration (*B*), and volume of extraction solvent per 0.3 g medicinal materials (*C*), with total peak area of SGR extracts (*Y*) as the response parameter. The Box-Behnken design experiment scheme is shown in Table S1.

As shown in Figure S1, the ultrasonic extraction twice is most efficient for extracting of bioactive ingredients in the SGR samples. On the basis of the single factor experiment, extraction time (*A*, 20–40 min), ethanol concentration (*B*, 40–80%), and volume of extraction solvent per 0.3 g medicinal materials (*C*, 6–8 mL) were chosen for further optimization. The RSM result indicated that the model was significant, and the influence order of three factors on the total peak area was B > A > C (Table S2, Figure S2). The optimal extractive conditions for SGR were as follows: ethanol concentration 71%, ultrasonic time 32 min, and liquid-to-material ratio of 30:1 mL/g (9 ml of extraction solvent was added to 0.3 g of medicinal materials for extraction), ultrasonic extraction twice. The model for total peak area in actual values was described in Equation :

*Y* = + 1214.6433 + 118.6926*A* + 72.7032*B* + 73.2870*C* – 0.4409*AB* –2.1002*AC* + 2.6317*BC* – 1.0639*A^2^* – 0.5803*B^2^* – 10.6119*C^2^*

In order to examine the suitability of the equation for predicting the optimal response value, the verification experiment was carried out, the result was in a good agreement with the predicted value, which was list in Table S3.

In summary, the optimal extractive conditions for SGR were as follows: ethanol concentration 71%, ultrasonic time 32 min, and liquid-to-material ratio of 30:1 mL/g (9 ml of extraction solvent was added to 0.3 g of medicinal materials for extraction).

**Table S1** The Box-Behnken design experiment scheme

| Std | Run | Factor 1  *A* | Factor 2  *B* | Factor 3  *C* | Response  *Y* |
| --- | --- | --- | --- | --- | --- |
| 15 | 1 | 30 | 60 | 8 | 6068.67 |
| 17 | 2 | 30 | 60 | 8 | 5973.4 |
| 13 | 3 | 30 | 60 | 8 | 5967.8 |
| 11 | 4 | 30 | 40 | 10 | 5343.13 |
| 14 | 5 | 30 | 60 | 8 | 5952.87 |
| 5 | 6 | 20 | 60 | 6 | 5636.53 |
| 12 | 7 | 30 | 80 | 10 | 6007.87 |
| 8 | 8 | 40 | 60 | 10 | 5909.25 |
| 7 | 9 | 20 | 60 | 10 | 5735.03 |
| 2 | 10 | 40 | 40 | 8 | 5607.52 |
| 10 | 11 | 30 | 80 | 6 | 5824.7 |
| 9 | 12 | 30 | 40 | 6 | 5581.03 |
| 1 | 13 | 20 | 40 | 8 | 5225.4 |
| 16 | 14 | 30 | 60 | 8 | 5855.94 |
| 4 | 15 | 40 | 80 | 8 | 5848.7 |
| 3 | 16 | 20 | 80 | 8 | 5819.33 |
| 6 | 17 | 40 | 60 | 6 | 5978.77 |

**Table S2** ANOVA of quadratic model terms of three factor variables on responses

| Source | Sum of Squares | df | Mean Square | F Value | p-value Prob > F | Inference |
| --- | --- | --- | --- | --- | --- | --- |
| Model | 8.723E+005 | 9 | 96922.65 | 26.31 | 0.0001 | significant |
| A | 1.076E+005 | 1 | 1.076E+005 | 29.22 | 0.0010 |  |
| B | 3.800E+005 | 1 | 3.800E+005 | 103.16 | < 0.0001 |  |
| C | 82.88 | 1 | 82.88 | 0.023 | 0.8850 |  |
| AB | 31109.61 | 1 | 31109.61 | 8.45 | 0.0228 |  |
| AC | 7057.40 | 1 | 7057.40 | 1.92 | 0.2088 |  |
| BC | 44324.28 | 1 | 44324.28 | 12.03 | 0.0104 |  |
| A^2^ | 47659.40 | 1 | 47659.40 | 12.94 | 0.0088 |  |
| B^2^ | 2.268E+005 | 1 | 2.268E+005 | 61.58 | 0.0001 |  |
| C^2^ | 7586.50 | 1 | 7586.50 | 2.06 | 0.1944 |  |
| Residual | 25783.02 | 7 | 3683.29 |  |  |  |
| Lack of Fit | 2924.55 | 3 | 974.85 | 0.17 | 0.9110 | not significant |
| Pure Error | 22858.47 | 4 | 5714.62 |  |  |  |
| Cor Total | 8.981E+005 | 16 |  |  |  |  |

**Table S3** Predicted and experimental values of the responses obtained under the optimal extraction conditions

|  | Extraction time(min) | Ethanol concentration (%) | Solvent volume (mL/0.3 g) | Total peak area |
| --- | --- | --- | --- | --- |
| Predicted value | 32.089 | 71.060 | 9.090 | 6035.257 |
| Experimental values(n=5) | 32 | 71 | 9 | 5974.4 |
| Relative error (%) |  |  |  | 1.01 |


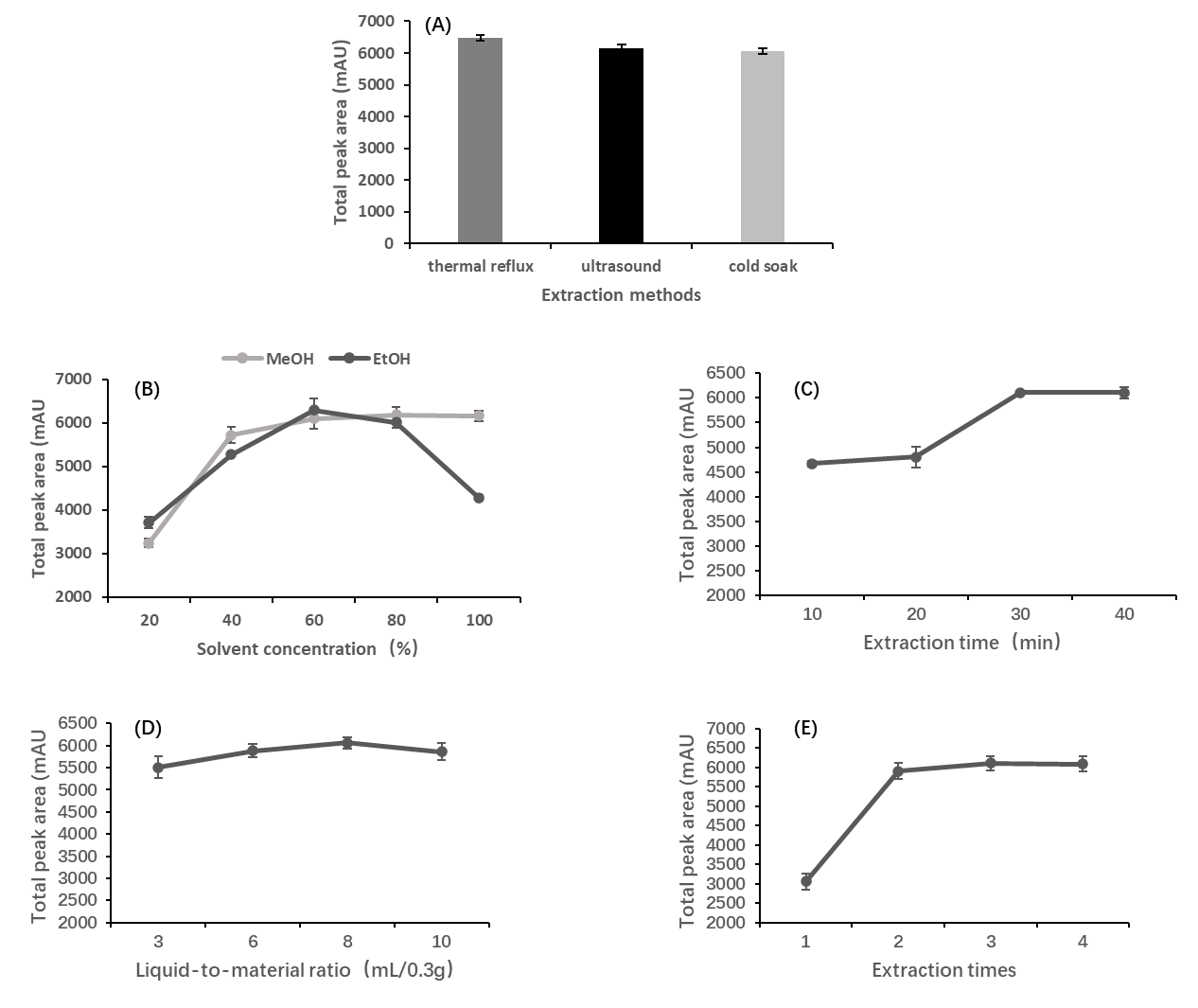


**Figure S1**. Result of the single factor experiments: (A) the effects of extraction methods on the total peak area; (B) the effects of solvent and concentration on the total peak area; (C) the effects of extraction time on the total peak area; (D) the effects of liquid-to-material ratio on the total peak area; (E) the effects of extraction times on the total peak area


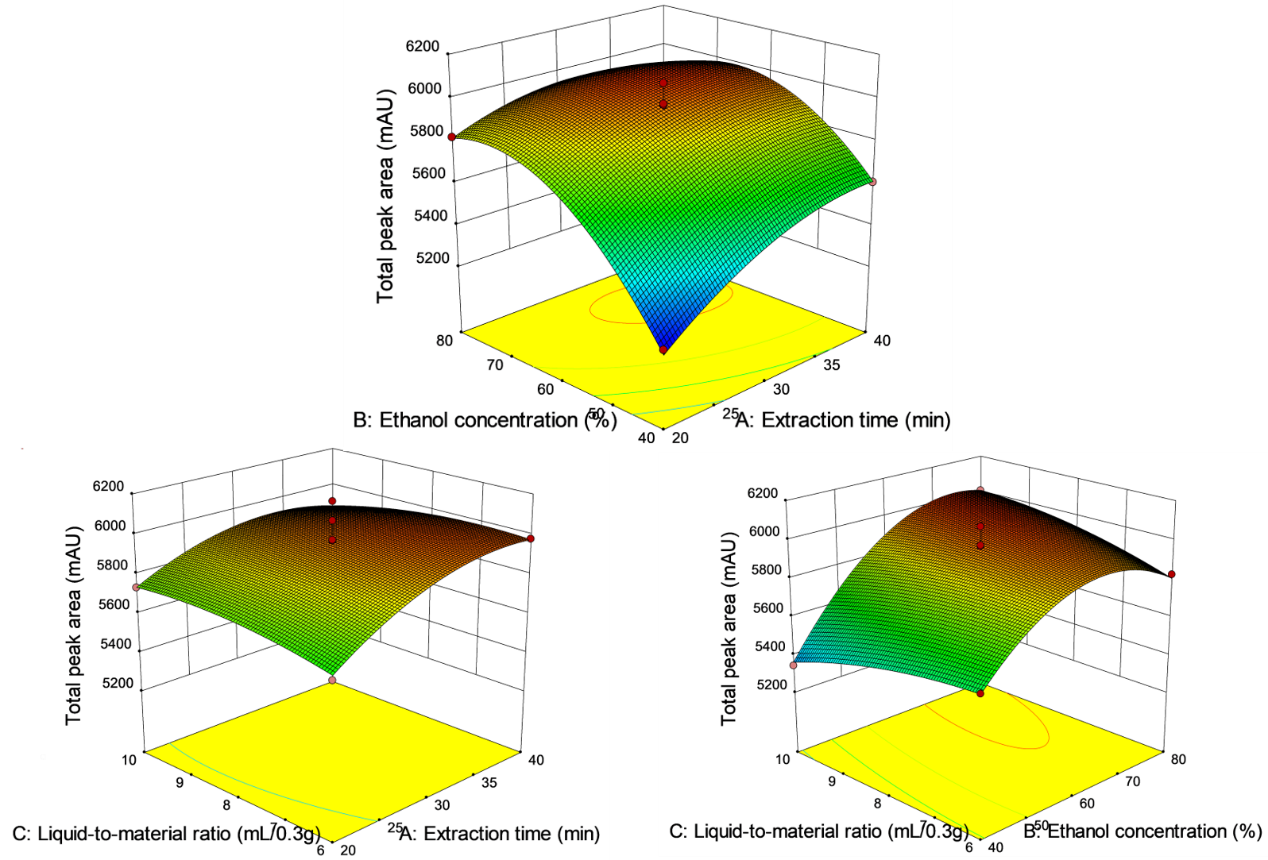


**Figure S2**. Response surface plots corresponding to the desirability function
